# Nursing supervision in the mobile pre-hospital emergency care service

**DOI:** 10.1590/1980-220X-REEUSP-2024-0238en

**Published:** 2025-07-04

**Authors:** Camila Galiano, André Almeida de Moura, Josué Souza Gleriano, Vivian Aline Mininel, Mayra de Cássia Trovó, Mariana Fraga de Figueiredo, Bethania Ferreira Goulart, Lucieli Dias Pedreschi Chaves

**Affiliations:** 1 Universidade de São Paulo, Escola de Enfermagem de Ribeirão Preto, Ribeirão Preto, SP, Brazil.; 2 Universidade de São Paulo, Escola de Enfermagem, Departamento de Orientação Profissional, São Paulo, SP, Brazil.; 3 Universidade do Estado de Mato Grosso, Enfermagem, Tangará da Serra, MT, Brazil.; 4 Universidade Federal de São Carlos, Enfermagem, São Carlos, SP, Brazil.; 5 Universidade Federal do Triângulo Mineiro, Enfermagem, Uberaba, MG, Brazil.

**Keywords:** Nursing, Supervisory, Nursing, Emergency Medical Services

## Abstract

**Objective:**

To analyze limitations and potentialities of nursing supervision, according to the nursing team, of a Mobile Emergency Care Service.

**Method:**

Descriptive research, with a qualitative approach, using the Critical Incident Technique. Nurses and nursing technicians participated. Data were collected in a semi-structured, individual, recorded interview, later transcribed, followed by the grouping and categorization of Critical Incidents, using Bardin’s content analysis.

**Results:**

Seventy-seven critical incidents emerged from the interviews, 22% received positive and 78% negative references, indicating a predominance of factors that limit supervision. These factors were categorized into “Singularities of nursing supervision”, “Organizational conditions”, “People management”, and “Vacancy regulation”.

**Conclusion:**

Enhancing factors: institutional support, education as a supervision tool, team meetings, timely feedback and participatory management; limiting factors: indirect nursing supervision (nurse and technicians in different teams), lack of materials and maintenance and of institutional support, nurse work overload, conflicts, and lack of communication.

## INTRODUCTION

The Brazilian Mobile Emergency Care Service (*SAMU*) is a public, complex service carried out within the context of the Emergency Care Network (*RUE*); among other activities, it is particularly characterized by its focus on pre-hospital emergency and urgency situations, with users’ care and referral for continuity of care, as well as critical patients transportation between health units^(1)^. Pre-hospital care in Brazil until the 1990s followed the North American model of Prehospital Care (PHC), with its organization varying in the different states; from 1997 onwards, regulations from the Ministry of Health (MS), influenced by the French model, consolidated mobile emergency care; and Ordinance MS 2048 of 2022 redefined the Brazilian system of urgent and emergency care, a mix of the North American and French models, consolidating *SAMU* ^(2)^.

*SAMU* uses different types of mobile units to respond to incidents, but essentially the land model is made up of: Basic Life Support Unit (*USB*), manned by the emergency vehicle driver and a nursing technician or assistant, and Advanced Life Support Unit (USA), manned by an emergency vehicle driver, a nurse, and a doctor^(3)^. This is an efficient strategy for first aid, stabilization, and transfer of patients to reference services. It is worth highlighting that nursing plays a relevant role in this service, whose constant evolution demands qualified professionals, capable of meeting health needs in pre-hospital emergency care, as well as in transportation to and between hospitals, with an emphasis on promoting, protecting, and restoring health, in addition to developing actions to ensure a good organizational climate and team satisfaction at work^(4)^.

Nurses play a central role, both in direct patient care and in team coordination, and their professional performance is regulated by specific legislation, such as Resolution of the Federal Nursing Council (COFEN) No. 713/2023, which updates the standards for action in mobile PHC, and the Opinion of the Regional Nursing Council of São Paulo (*COREN-SP*) No. 005/2019, which defines the profile and skills of the Nursing Technician in this scenario. These laws reinforce the importance of nurses in resource management, decision-making, and emergency regulation, which are essential for the effectiveness of pre-hospital care, in line with the guidelines established by *SAMU* to ensure quality and safety in patient care^(5,6,7)^.

In coordinating the care process, nursing supervision identifies specific care and management needs, to establish plans and goals that contribute to a comprehensive approach to the patient^(8)^. It is important to note that the nurse has the exclusive responsibility of supervising the nursing team, in an educational process of integration and coordination, which results in qualified care for the needs of patients and institutions^(9)^.

Nursing supervision can be understood from different perspectives, as a dynamic management instrument, which has long faced the challenge of advancing in forms of performance that guarantee the adequate production of care actions, from the perspective of comprehensiveness and in a timely manner, but with a more participatory, collaborative, intergenerational approach^(10)^. Supervision may focus on controlling the organization of work, the internal standardization of health services, as well as the possibility of ensuring that what is necessary is produced, at the appropriate time and in the appropriate manner; it may have an educational perspective of supervision that encompasses the active participation of the team, through educational actions/practices, as well as having focus on political articulation regarding the role of supervision as a mediator between institutional policy and the team, to make nursing care viable^(11)^.

This study is justified by the peculiarity of the exercise of nursing supervision in *SAMU*, which requires agility in decision- making processes and a comprehensive approach to patients’ needs^(11,12)^, added to the fact that the nurse and the nursing technician are not in the same physical space during care^(7)^. Furthermore, scientific research is essential to understand the complexity of organizational and procedural aspects related to supervision, the results of which allow the development of strategies to support nurses, to enhance the qualification of the care provided^(8,13)^ and the last national work on the specificity of nursing supervision in *SAMU* was published a decade ago^(13)^. In this sense, the question is: Which factors facilitate and which hinder nursing supervision in *SAMU*? Given the above, the study aims to analyze limitations and potentialities of nursing supervision, according to the nursing team of a *SAMU*.

## METHOD

### Design of Study

Descriptive research, with a qualitative approach, using the Critical Incident Technique (CIT), which requires compliance with the following steps: Determination of the objectives of the activity to be performed; Preparation of questions to be asked to the people who will provide the critical incidents of the activity to be analyzed; Population delimitation; Collection of critical incidents; Analysis of the content of the incidents collected; Grouping and categorization of critical incidents; Survey of the frequencies of positive and negative critical incidents^(8,14)^.

It should be noted that this work followed the recommendations of the international protocol Consolidated Criteria for Reporting Qualitative Research (COREQ)^(8,15)^ for presenting research results.

### Study Local

The research was carried out at a decentralized *SAMU* base, in a municipality in an inland city of the state of São Paulo, with an estimated population of 127 thousand inhabitants, in 2022, distributed over approximately 400 km^2^ of territory^(16)^. The service has two USBs that serve residents and a USA that serves the region and residents.

### Population and Selection Criteria

The participants were nurses (ENF, for the Portuguese word *Enfermeiro*) and nursing technicians (TE, *Técnicos de enfermagem* in Portuguese) from *SAMU*, totaling seven nurses and fourteen nursing technicians, 21 potential participants. The inclusion criteria used were: professionals working in the service investigated for at least one year, a period of time that allows the professional to have experience in inserting themselves into the unit’s activities.

### Data Collection

An interview script was organized to collect data, aimed at meeting the CIT recommendations^(8,14)^, consisting of three topics: participant identification; two open questions, with similar text, one with a positive reference and the other with a negative reference, about situations experienced or observed regarding nursing supervision at *SAMU*, how people behaved and the consequences arising from the situation, in addition to another open question asking participants for suggestions to improve nursing supervision at *SAMU*.

The script underwent face validation by judges, nurse-teachers, intentionally chosen due to their mastery of the topic and method, and was submitted to pre-testing, with professionals who perform the same function as the participants, but in units different from those of the collection, to ensure the quality and applicability of the interview script in real conditions. It should be noted that adjustments were made to the form and not to the content for the final version of the script. Data collection took place from April to June 2022, through semi-structured, in-person and individual interviews, in a private environment, which lasted approximately 30 minutes and were conducted by the study’s main researcher, after participants’ consent.

### Data Analysis and Treatment

The interviews were recorded on two digital devices, transcribed in full by the researcher and checked with the respective audios by another researcher, being stored in a database of an institutional e-mail of the research center. To avoid identifying the participants, the statements were coded with “ENF” for nurse or “TE” for nursing technician, followed by the letter “E” for Interview (*entrevista* in Portuguese) and subsequently added by numbers (1, 2, 3.... up to 17) in the sequential order in which they were carried out, representing each of the participants.

The main researcher systematized the data and, considering the CIT analysis, extracted the situations, behaviors, and consequences, composing the CIs, which received, from the participants’ perspective, positive or negative references, corresponding to the potentialities and limitations of nursing supervision, respectively. Descriptive statistics was initially used to quantify the CIs and perform content analysis^(17)^.

Data were analyzed using Bardin’s content analysis^(18)^, submitted to pre-analysis, with thorough reading and preparation of the material, including grammatical corrections, excluding language vices, interferences and external noises, aiming to allow the reader to understand the meaning of the interview; subsequently, the material was explored with the reports pertinent to each situation, behavior and consequence, grouped by similarity of content; and finally, the results obtained were interpreted according to the context of *SAMU* and theoretical assumptions of nursing supervision.

### Ethical Aspects

The study was assessed and approved by the Research Ethics Committee of the Ribeirão Preto School of Nursing at the Universidade de São Paulo, according to opinion no. 4.240.326 (on August 27, 2020), to meet the requirements of Resolution No. 466/2012 of the National Health Council. All participants signed the Free and Informed Consent Form before doing the interviews.

## RESULTS

Seventeen professionals were interviewed, divided between ten nursing technicians (59%) and seven nurses (41%). Of the total of 21 SAMU nursing professionals, four did not meet the inclusion criteria. The majority of interviewees were female (n = 10; 59%). There was a greater concentration of participants in the age group of 41 to 50 years (n = 8; 47%); between 10 and 15 years of professional training (n = 7; 41%), who worked in the unit between six and ten years (n = 7; 41%).

From the 17 interviews, 77 CIs were generated, of which 17(22%) received positive and 60(78%) negative references, grouping these CIs into four categories: Singularities of nursing supervision, with 34% positive references and 66% negative references; Organizational Conditions, with 100% negative references; People management with 20% positive references and 80% negative references and Vacancy regulation, with 12% positive references and 88% negative references.

### Singularities of Nursing Supervision

The category “Singularities of nursing supervision” groups CIs related to the characteristic mode of nursing supervision in *SAMU*, in which technicians and nurses belong to different teams. There was a predominance of incidents with negative references, as it can be seen:

*I don’t know what the technician is doing, unless I’m with him or it’s a very discrepant problem that I hear on the radio, but I’m not listening to the radio all the time; the supervision here is not direct, it’s at a distance, the nurse doesn’t have contact with the nursing technician all the time* (ENF E14).

*Sometimes the technician is doing something wrong; for example, he or she wasn’t performing cardiac massage correctly, but he or she would never ask me if I wasn’t there, monitoring the procedure.* (ENF E01)

*Here at SAMU, supervision is very indirect, the nurse cannot be with the nursing technician in the ambulance all the time, they are separate vehicles, we are not there at the exact moment of care to supervise, guide a technique, care* (ENF E06).

In contrast, ways to minimize the negative aspects are suggested, with CIs representing positive perspectives (34%):

*What helps most, when there are problems with care, is being able to clarify doubts with nursing supervision, to complement, in the sense of adding, you know? But most of the time, they don’t conduct direct supervision.* (TE E7)

*I talk a lot with the team about the care I take part in, I am interested in what they do; when there is something different or something they discussed, I want to know the details of what happened; this interaction favors nursing supervision* (ENF E14)

*The habit of arriving at the base and discussing the care, both with the nurse and among us, technicians and first responders, is always a positive thing to reflect on and learn from.* (TE E10)

Regarding the uniqueness of nursing supervision, the absence of direct supervision proves to be a limiting factor, which arises from the specificity of the work process in decentralized *SAMU* bases.

### Organizational Conditions

The “Organizational Conditions” category groups incidents involving the cleaning and maintenance of mobile service units and the SAMU base, management and availability of materials and work supplies.

*When there is a problem, people from USB end up with an inferior ambulance, because USA cannot work with an inferior ambulance, but the basic one can, but it is not good for the service.* (TE E07)

*Cleaning and hygiene are complicated here, most of the staff don’t clean and I don’t see supervision taking action.* (TE E11)

*I took over a shift and didn’t have time to complete the ambulance checklist; I went out to provide care and considered that my colleague had left everything in order, so I could go in and work. At the scene, there was a patient with BIPAP, who needed to be connected to the oxygen network to avoid desaturation, and the oxygen cylinder was empty.* (TE E17)

*They don’t always sanitize the ambulance, and I don’t see nursing supervision continually working to do so.* (ENF E9)

It is worth noting that there were no positive aspects in the “Organizational Conditions” incident category.

### People Management

The “People Management” category groups together incidents related to team sizing, shift management, and conflict management. It presented a higher frequency of negative incidents:

*The nurse cannot authorize work extra hours, but needs to change shifts, there are many Sick Leaves and the service has to be viable.* (TE E3)

*When I passed the exam to work at the city hall health department, I was informed that I would work at SAMU, which had a vacancy available, but I wanted to work in another type of health service.* (TE E16)

*I have witnessed situations where an employee wanted to attack another, fights due to the delay in responding to a request, conflicts that are generated by some people’s type of personality.* (ENF E15)

However, positive incidents were also highlighted:

*During the pandemic, we were able to have two nurses on duty; it was possible to go out to see the technician together, which made a big difference: being able to teach, monitor what is being done and how it is being done.* (ENF E6)

*Covid, despite having brought so much pain, brought some good things in terms of updating knowledge for the nursing team.* (ENF E1)

### Vacancy Regulation

The “Vacancy regulation” category brought together ICs referring to the relationship between nursing professionals and the demands of the Regulation Center (CR) for *SAMU*, with a predominance of negative incidents:

*The CR is outside the municipality, it is regional and the regulatory staff do not know the local reality very well and this complicates the work.* (ENF E1)

*A very difficult type of discussion that happens is between the unit crew and the CR staff; the nurse is in this middle ground, having to guide so as not to give rude answers or question the type of care, but the USBs always question and there is a dispute with the CR.* (ENF E14)

*CR calls for very low complexity services, I would say “banal” services* (TE E12)

*You’re in the car, if you’re not and something happens to the patient, you know what my position is! Go, do it and, when you arrive, report what is happening; do your best, use your last resort, everything you can, and then we can question and talk to the CR about anything later.* (ENF E14)

## DISCUSSION

Regarding the participants’ sociodemographic and professional variables, it can be seen that the results are similar to the findings of work developed in the same context, in the metropolitan region of São Paulo^(19)^. Skills are developed over years of professional practice, and it takes nurses two to three years to achieve adequate practical performance, which generally results from successive approaches to everyday problems and possible solutions^(20)^.

In basic care, the TE is not with the nurse at all times, which makes nursing supervision challenging, as it hinders the assessment and support from the nursing team when providing patient care. Nursing supervision should be conducted as a dynamic process, based on situational analysis, followed by the implementation of interventions to achieve the established objectives – which should be reassessed based on the results obtained – and to make adjustments, if necessary^(21)^.

The category “Supervision singularities” had a predominance of incidents that limited the performance of nursing supervision. The characteristics of the work process, in which, for basic care, the TE is not with the nurse all the time, make nursing supervision peculiar at *SAMU*. Overcoming barriers related to nursing supervision is multifactorial; there are intentions and issues highlighted in daily work practice^(10)^. The use of supervision under an educational approach is powerful, but requires institutional support and investment from the nurse in developing the team’s autonomy.

The results show that the participants indicated paths that favored nursing supervision – with special emphasis on meetings after care – paths that were built collectively and horizontally, which have a positive impact on work and favor reflection on practice, provide opportunities for the exchange of knowledge, and can direct initiatives that provide more robust theoretical support. Education stands out as a tool for nursing supervision, carried out based on timely feedback from the nurse to the TE team and from the nursing coordinator to the nurses. A clear and constructive feedback aims to improve communication, increase confidence and security in professional performance^(8)^. Creating spaces and opportunities for empathetic listening and using measures that encourage interaction between team members are essential actions as a way of qualifying the team and overcoming challenges^(10)^.

In the “Organizational Conditions” category, only negative incidents were listed, that is, those that limit nursing supervision. It is worth noting that this category groups together incidents that commonly transcend the nurse’s power of deliberation, since providing adequate structure, supplies, and other organizational aspects – so that care and management processes can take place fully – is an institutional responsibility. However, even so, the responsibility for actions related to the organization falls on this professional, which corroborates their role as a reference in the service.

It should be noted that insufficient infrastructure, materials, and maintenance are reasons for dissatisfaction among professionals and weaken assistance, as well as – added to the lack of scale/organization for activities inherent to the routine, such as cleaning, organization and replacement of materials – cause the team to work in a disjointed and unmotivated manner, giving the false impression that these are activities of lesser importance, but are essential to guarantee adequate and safe care in the event of incidents. Inadequacy and insufficiency of materials, equipment and support vehicles – aspects related to institutional support for the functioning of the service – are not within the nurse’s control, but have repercussions in conditions that may represent care and professional risks, bringing feelings of insecurity and devaluation.

Actions such as the maintenance of life support vehicles are essential for safe and efficient care, since basic and advanced support units are the team’s workplace until the user reaches the reference service^(22)^. The inadequacy of maintenance of basic and advanced support units can expose patients and the health unit itself to unsafe conditions, which may result in ethical and legal sanctions for the institution^(23)^.

The implementation of schedules for daily activities, such as checking materials, organization and cleaning, allows the team to value the activity, so that it achieves the purpose aim of controlling nursing supervision, providing standardization to the service. Furthermore, occupational stress perceived by emergency nurses is worsened by the scarcity of resources; low support provided; poor definition of roles and responsibilities; lack of autonomy and excessive workload^(24)^.

The development of nursing supervision also has to consider the context and conditions in which the work of the nursing team and of the nurse is being developed; it is unlikely to achieve excellent practices in flawed organizational conditions; in such scenarios, nursing supervision is often reduced to a dimension of production control. The exercise of supervision in services benefits the team with an increased feeling of support, favors the reduction of emotional fatigue, added to the improvement in the relationship between the nursing team, which can reflect in an improvement in job satisfaction and in the development of professional practices^(25)^.

In the “People management” category, there was a predominance of negative incidents. Difficulties in communication and presence of conflicts, both within the team and between support bodies, interfere in the process of nursing supervision practice and impact professional satisfaction and the performance of daily activities. A lack of objectivity and clarity in communication can result in inappropriate planning for responding to the incident. Furthermore, the lack of specific guidance for hiring personnel for a given service may result in dissatisfied professionals, as they are unable to be placed in the workplaces with which they have the greatest affinity.

In this regard, it is understood that supervision and communication are essential management skills for nurses, in leading the team, managing conflicts, and achieving the amplitude of professional practice in *SAMU*, exercising leadership in a democratic and horizontal manner, supported by technical and scientific knowledge^(26)^. Adversities inherent in interpersonal relationships must be faced beyond the search for a humanized, ethical, and collaborative daily practice, but also to minimize or even avoid the legitimization of evasive and defensive means in the face of challenging situations in daily work^(10)^, to promote quality in care and preserve workers’ psychological well-being. The quality of the environment and work is essential, especially in the pandemic scenario^(27)^.

In the context of SAMU, changes resulting from the pandemic resulted in an increase in the number of professionals, a factor that enhanced nursing supervision, allowing the opportunity to experience positive developments from direct supervision and care in conjunction with the TE. However, it is important to highlight that changes resulting from the pandemic have intensified the challenges that already existed in SAMU’s operations^(28)^.

The sizing of the nursing team, below the needs of the service, impacts the quality of care in the different areas of nursing activity, generates work overload, favors stress, the occurrence of errors and adverse events, factors that compromise patient safety^(29)^.

The promotion of educational actions from the perspective of continuing and permanent education and the organization of safe environments among the team, for the discussion of cases with a theoretical basis for nursing supervision, in line with technical knowledge – as well as periodic meetings among nurses, to improve professional practice, exchange experiences on managerial and organizational issues, establishing environments for collective construction, encouraging the internal strength of the team – were considered factors that enhance nursing supervision in this work context. A supportive environment among professionals for exchanging experiences, communication, and promoting training on topics relevant to the context is a positive contribution to the team as a whole and, consequently, an improvement in the qualification of care. This support includes protocols and assistance techniques, cleaning, use of equipment and technologies linked to direct care, as well as discussions of cases after occurrences, already present in the service, which consider reflections on situations experienced.

Support for the inclusion of educational activities (courses and training) – carried out during working hours, without harm to the worker and with adequate staffing to cover the work schedule – could be a way of facing the challenges inherent to the practice of nursing supervision at *SAMU*. By analyzing opportunities for improvement, it becomes possible to boost the development of professionals’ skills through the practical application of knowledge^(30)^.

The “Vacancy regulation” category concentrated the largest number of negative references, which represents limitations for nursing supervision. Incidents related to the vacancy regulation center can be understood as complex, since – although they are a critical interface between the health system and services with users and professionals – they often trigger communication problems and conflicts, which have repercussions on user satisfaction and adequacy of care, on the attributions and responsibilities of different points in the care network and on the performance of the respective health teams. Particularly for nursing, it can be inferred that it represents the dimension of the political articulation of supervision.

*SAMU’s* professionals depend directly on the guidance and proper functioning of the Emergency/Urgent Regulation Center (CRUE) to carry out their work; on the other hand, they also do not have visibility over the availability of vacancies and the entire health context of the region, at the time of care. Therefore, it is necessary to establish a relationship of trust, with the purpose of promoting agile, safe, and efficient care for the patient, minimizing health problems.

Some *SAMU* users are unaware of or have not been informed about the health service; they should seek the reason why they choose the care they consider appropriate for the problem faced at that time. It is common to use this service as a means of transport, to solve social demands, or as a gateway to the Brazilian Public Health Service, outside of emergency situations, as it is a service in which the user will quickly find a complete team of professionals, immediate treatment, and more complex services^(22)^.

The above highlights macro-organizational aspects, which also have repercussions on nursing supervision; for example, noise in the interface with the CRUE, which can cause dissatisfaction among professionals, results in conflicts and delays in care, conditions that are independent of the direct action of *SAMU* professionals. In this sense, coping strategies cannot be simplistic; however, the ability to use educational strategies with the nursing team to mitigate communication problems and conflicts, as well as the opportunity for the team to inform the population about the use of *SAMU* and other health services, are highlighted in a timely manner.

As a limitation of the study, the analysis of only one service is highlighted, which restricts the possibility of a broader analysis of the object of study; furthermore, at the time, the relaxation of security measures related to the Covid-19 pandemic was beginning and a process of readjustment of health services was underway. However, despite the limitations, the study contributes significantly to the field of nursing, by emphasizing aspects that limit and enhance nursing supervision, providing valuable insights for improving professional practice and creating an environment of learning and dialogue. Finally, the intention is not to propose a guide or work model, but the results allow suggesting aspects to be considered in the approach regarding nursing supervision in SAMU, such as the possibility of exploring the practice of direct supervision whenever possible, with the nurse as a member of the basic support team together with the nursing technician.

Based on the results and discussion, a summary of aspects that enhance the implementation of nursing supervision at SAMU is presented: institutional support regarding organizational aspects such as transportation vehicles, cleaning materials and maintenance, and regarding adjustment instruments to meet emergency shift demands, such as extra hours; support for the inclusion of educational activities during working hours, without harm to the worker, using education as a tool for nursing supervision, based on timely feedback; encouragement of a supportive environment among professionals, aiming to stimulate exchanges of experiences; encouragement of discussions of cases after occurrences, to consider reflections on situations experienced, analyzed and guided by technical-scientific and ethical knowledge; periodic meetings with the team, as opportunities for exchanging knowledge, with clear and objective, horizontal communication, capable of reflecting on the appreciation of professionals and their functions; adequate sizing of the professional staff to cover the work schedule together with the organization of the service for daily activities, based on tools such as work schedules, protocols and operational procedures for cleaning routines, checking and replacement of materials; encouragement and conduction of direct supervision of the nurse during the care of the BLS team, whenever possible, with the objective of supporting, evaluating professional practice, and carrying out necessary interventions during practice or through educational measures after care.

Still considering the results and discussion, the summary of limiting factors for the performance of nursing supervision in *SAMU* is presented: The work process itself directs the team based on the type of care, which results in team fragmentation, since, in the Basic Life Support (BLS) team’s care, the TE and the nurse do not occupy the same space, which makes nursing supervision challenging in this service, because there is no possibility of evaluation and support from the nursing team at the time of patient care. Regarding organizational aspects, the inadequacy and insufficiency of materials, equipment and support vehicles maintenance are not within the nurse’s control, but have repercussions in conditions that may represent care and professional risks; also on this topic, the lack of organization for activities inherent to the routine, such as cleaning, organization and replacement of materials, causes the team to work in a disjointed and unmotivated manner and impacts the adequate and safe care of occurrences. Added to these factors are communication difficulties and conflicts present both within the team and between support bodies.

## CONCLUSION

Based on the results achieved in this research, it became possible to identify the predominance of aspects that limit the performance of nursing supervision in the context of SAMU. This highlights the urgency of a closer look – particularly with regard to nursing supervision – at the critical requirements needed to perform this activity and the insufficient educational approach dedicated to this managerial component, in the context studied.

The results encourage reflection on the performance of the nurse’s role in exercising supervision, but also reveal relevant aspects related to care management at *SAMU*. This scenario contributes significantly to identifying and improving critical points, providing support to nurses in terms of service management, team management and provision of assistance. In this context, even if the results are not generalizable, it is plausible that other units in similar contexts may benefit from the investigations presented in this study.

There is much to be done in theoretical and practical knowledge about the exercise of nursing supervision at *SAMU*, especially from an educational perspective, which, even though considered a resource used, is still limited in its potential.

## DATA AVAILABILITY

The dataset supporting the findings of this study is not publicly available.

## References

[1] Malvestio MAA, Sousa RMC (2024). Produção de procedimentos pelo SAMU 192 no Brasil: performance,
benchmarking e desafios. Cien Saude Colet.

[2] Martins PPS, Prado ML (2003). Enfermagem e serviço de atendimento pré-hospitalar: descaminhos e
perspectivas. Rev Bras Enferm.

[3] Malvestio MAA, Sousa RMC (2022). Desigualdade na atenção pré-hospitalar no Brasil: análise da
eficiência e suficiência da cobertura do SAMU 192. Cien Saude Colet.

[4] Teixeira EP, Araújo AHIM (2023). O atendimento de enfermagem no SAMU e seu respaldo legal: revisão
bibliográfica. Rev JRG Est Acad.

[5] Brasil. (2022). Conselho Federal de Enfermagem – COFEN. Resolução COFEN nº 713, 4 de
novembro de 2022. Diário Oficial da União [Internet].

[6] São Paulo. (2019). Conselho Regional de Enfermagem de São Paulo – COREN-SP. Parecer
normativo nº 005/2019. Perfil do Responsável Técnico de Enfermagem nos
serviços de atendimento pré-hospitalar. Diário Oficial do Estado
[Internet].

[7] Andrade TF, Silva MMJ (2019). Características dos enfermeiros no atendimento pré-hospitalar:
concepções sobre a formação e exercício profissional. Enferm Foco.

[8] Rocha IARS, Rodrigues MAC, Pinto CMCB, Carvalho ALRF (2021). Supervisão clínica em enfermagem para otimizar a avaliação do
autocuidado. Cogitare Enferm.

[9] Xavier-Gomes LM, David GF, Tibães HBB, Frison SS, Andrade-Barbosa TL (2015). Vislumbrando “O Artífice” no cotidiano de trabalho das
enfermeiras na gerência hospitalar. Mundo Saude.

[10] Giacomini MA, Chaves LDP, Galiano C, Alves LR, Mininel VA, Henriques SH (2022). Supervisão de enfermagem: instrumento gerencial de qualificação
da equipe e do cuidado. Rev Enferm UFSM.

[11] Moura AA, Bernardes A, Balsanelli AP, Dessotte CAM, Gabriel CS, Zanetti ACB (2020). Leadership and job satisfaction in the Mobile Emergency Care
Service context. Rev Lat Am Enfermagem.

[12] Pereira AB, Martins JT, Ribeiro RP, Galdino MJQ, Carreira L, Karino ME (2020). Work weaknesses and potentials: perception of mobile emergency
service nurses. Rev Bras Enferm.

[13] Bernardes A, Maziero VG, Hetti LBE, Baldin MCS, Gabriel CS (2014). Supervisão do enfermeiro no atendimento pré-hospitalar
móvel. Rev Eletr Enferm.

[14] Ribeiro LCM, Souza ACS, Barreto RASS, Neves HCC, Barbosa MA (2012). Critical incident technique and its nursing use: an integrative
literature review. Rev Bras Enferm.

[15] Souza VRS, Marziale MHP, Silva GTR, Nascimento PL (2021). Translation and validation into Brazilian Portuguese and
assessment of the COREQ checklist. Acta Paul Enferm.

[16] Instituto Brasileiro de Geografia e Estatística. (2020). Censo demográfico: resultados preliminares: Sertãozinho
[Internet].. https://cidades.ibge.gov.br/brasil/sp/sertaozinho/panorama.

[17] Coleta JAD (1974). A técnica dos incidentes críticos: aplicações e
resultados.. Arq Bras Psicol Apl..

[18] Bardin L (2011). Análise de conteúdo..

[19] Ribeiro JB, Santos JJ, Almeida HOC, Lima DM, Melo IA (2021). Qualidade da assistência intervencionista prestada pelo serviço
de atendimento móvel de urgência em Aracaju Sergipe, Brasil. Interfaces Científicas Saúde e Ambiente.

[20] Warshawsky N, Cramer E (2019). Describing nurse manager role preparation and competency:
findingsfrom a national study. J Nurs Adm.

[21] Pereira AB, Martins JT, Ribeiro RP, Galdino MJQ, Carreira L, Karino ME (2020). Work weaknesses and potentials: perception of mobile emergency
service nurses. Rev Bras Enferm.

[22] Fernandes SC, Pinto VMP, Seara MLP S, Gomes LMRM, Clemente FAM, Santos MJE (2024). Estratégias supervisivas dos estudantes de enfermagem em ensino
clínico.. Rev Cienc Enferm..

[23] Lumer S, Oliveira GO, Diniz FJL S (2018). Central de regulação de urgências e emergências: possibilidades e
dificuldades da regulação médica na cidade do Rio de
Janeiro.. Rev Gest Saúde..

[24] Montero-Tejero DJ, Jiménez-Picón N, Gómez-Salgado J, Vidal-Tejero E, Fagundo-Rivera J (2024). Factors influencing occupational stress perceived by emergency
nurses during prehospital care: a systematic review. Psychol Res Behav Manag.

[25] Nuritasari R, Rofiqi E, Fibriola T, Ardiansyah R (2019). The effect of clinical supervision on nurse
performance.. Jurnal Ners [Internet]..

[26] Almeida DB, Silva GTR, Brasileiro DLS, Santos NVC, Santana LS, Santos CM (2023). Identidade profissional da enfermeira no Serviço de Atendimento
Móvel das Urgências. Hist Enferm Rev Eletr.

[27] Unal M, Yilmaz A, Yilmaz H, Tasdemir GY, Uluturk M, Kemanci A (2022). The impact of COVID-19 on social support perception and stress of
prehospital care providers. Australas Emerg Care.

[28] Rodrigues MPB, Silva ACC, Duarte GP, Pitanga KK, Traboulsi LS, Silva CTX (2023). Efeitos da pandemia do novo coronavírus no serviço de atendimento
móvel de urgência (SAMU) nas ocorrências em uma cidade do interior de
Goiás. Evidência.

[29] Vicente C, Amante LN, Sebold LF, Girondi JBR, Martins T, Salum NC (2021). Dimensionamento de enfermagem em unidade de internação cirúrgica:
estudo descritivo. Cogitare Enferm.

[30] Zacarias SP, Maximiniano ACA (2023). Fatores críticos de sucesso para o desenvolvimento executivo em
administração de projetos: uma proposta de inovação nas práticas docentes em
cursos de pós-graduação. In: Anais do Encontro de Estudos em Estratégia -
3Es [Internet].

