# Supplementary material for: Nursing supervision in the mobile pre-hospital emergency care service
Source: Rev Esc Enferm USP. 2025 Jul 4;59:e20240238. doi: 10.1590/1980-220X-REEUSP-2024-0238en (PMC12244992; doi:10.1590/1980-220X-REEUSP-2024-0238en)
Supplement: Translation

# Translation: Supervisão de enfermagem no serviço de atendimento pré-hospitalar móvel de urgência

**DOI:** 10.1590/1980-220X-REEUSP-2024-0238pt

**Authors:** Camila Galiano, André Almeida de Moura, Josué Souza Gleriano, Vivian Aline Mininel, Mayra de Cássia Trovó, Mariana Fraga de Figueiredo, Bethania Ferreira Goulart, Lucieli Dias Pedreschi Chaves

**Affiliations:** 1 Universidade de São Paulo, Escola de Enfermagem de Ribeirão Preto, Ribeirão Preto, SP, Brasil.; 2 Universidade de São Paulo, Escola de Enfermagem, Departamento de Orientação Profissional, São Paulo, SP, Brasil.; 3 Universidade do Estado de Mato Grosso, Enfermagem, Tangará da Serra, MT, Brasil.; 4 Universidade Federal de São Carlos, Enfermagem, São Carlos, SP, Brasil.; 5 Universidade Federal do Triângulo Mineiro, Enfermagem, Uberaba, MG, Brasil.

**Keywords:** Supervisão de Enfermagem, Enfermagem, Serviços Médicos de Emergência

## Abstract

**Objetivo:**

Analisar limitações e potencialidades da supervisão de enfermagem, segundo a equipe de enfermagem, de um Serviço de Atendimento Móvel de Urgência.

**Método:**

Pesquisa descritiva, de abordagem qualitativa, utilizando Técnica do Incidente Crítico. Participaram enfermeiros e técnicos de enfermagem. Os dados foram coletados em entrevista semiestruturada, individual, gravada, posteriormente transcrita, seguida do agrupamento e categorização dos Incidentes Críticos, utilizando análise de conteúdo de Bardin.

**Resultados:**

Das entrevistas, emergiram 77 Incidentes Críticos, 22% receberam referência positiva e 78% negativas, indicando predomínio de fatores que limitam a supervisão. Esses fatores foram categorizados em “Singularidades da supervisão de enfermagem”, “Condições Organizativas”, “Gestão de pessoas” e “Regulação de vagas”.

**Conclusão:**

Fatores potencializadores: apoio institucional, educação como ferramenta de supervisão, reuniões de equipe, “*feedback”* oportuno e gestão participativa; fatores limitantes: supervisão de enfermagem indireta (enfermeiro e técnicos em equipes distintas), falta de materiais e de manutenção e apoio institucional, sobrecarga de trabalho do enfermeiro, conflitos e falta de comunicação.

## INTRODUÇÃO

O Serviço de Atendimento Móvel de Urgência (SAMU) é um serviço público, complexo e realizado no contexto da Rede de Atenção às Urgências e Emergências (RUE); dentre outras atividades é, particularmente, caracterizado pelo foco nas situações de urgências e emergências pré-hospitalares, com atendimento e encaminhamento dos usuários para continuidade da assistência, além de realizar o transporte de pacientes críticos entre unidades de saúde^(1)^. O atendimento pré-hospitalar no Brasil, até a década de 1990, seguia o modelo norte-americano de Atendimento Pré-Hospitalar (APH), variando sua organização nos diferentes estados; a partir de 1997, normatizações do Ministério da Saúde (MS), influenciadas pelo modelo francês, consolidaram o atendimento móvel de urgência; e a Portaria MS 2048, de 2022, redefiniu o sistema brasileiro de atenção às urgências e emergências, misto dos modelos norte-americano e francês, consolidando o SAMU^(2)^.

O SAMU utiliza diferentes tipos de unidades móveis para atendimento das ocorrências, mas essencialmente o modelo terrestre é composto pela Unidade de Suporte Básico (USB) de vida, tripulada pelo condutor do veículo de urgência e um técnico ou auxiliar de enfermagem e Unidade de Suporte Avançado (USA) de vida, tripulada por um condutor de veículo de urgência, um enfermeiro e um médico^(3)^. Trata-se de estratégia eficiente de primeiros socorros, estabilização e deslocamento de pacientes para serviços de referência. Cabe destacar que a enfermagem tem um papel relevante nesse serviço, cuja evolução constante demanda profissionais qualificados, capazes de atender às necessidades de saúde no cuidado pré-hospitalar de urgência, bem como no transporte para e entre hospitais, com ênfase na promoção, resguardo e restauração da saúde, além de desenvolver ações para assegurar o bom clima organizacional e satisfação da equipe no trabalho^(4)^.

O enfermeiro desempenha um papel central, tanto na assistência direta ao paciente quanto na coordenação das equipes, e a sua atuação profissional está regulamentada por legislações específicas, como a Resolução do Conselho Federal de Enfermagem (COFEN) nº 713/2023, que atualiza as normas de atuação no APH móvel, e o Parecer do Conselho Regional de Enfermagem de São Paulo (COREN-SP) nº 005/2019, que define o perfil e as competências do Técnico de Enfermagem nesse cenário. Essas legislações reforçam a importância do enfermeiro no gerenciamento de recursos, na tomada de decisões e na regulação das urgências, essenciais para a eficácia do atendimento pré-hospitalar, alinhando-se às diretrizes estabelecidas pelo SAMU para assegurar a qualidade e a segurança no cuidado ao paciente^(5,6,7)^.

Na coordenação do processo assistencial, a supervisão de enfermagem identifica necessidades assistenciais e gerenciais específicas, para estabelecer planos e metas que contribuem para uma abordagem integral ao paciente^(8)^. Ressalta-se que o enfermeiro tem a atribuição privativa de supervisionar a equipe de enfermagem, em um processo educativo de integração e coordenação, que resulta em atendimento qualificado das necessidades dos pacientes e instituições^(9)^.

A supervisão de enfermagem pode ser entendida sob diferentes perspectivas, como um instrumento gerencial dinâmico, que há muito vivencia o desafio de avançar em formas de desempenho que garantam a produção adequada de ações cuidativas, na perspectiva da integralidade e em tempo oportuno, mas com abordagem mais participativa, colaborativa, intergeracional^(10)^. A supervisão pode ter enfoque de controle da organização do trabalho, da padronização interna dos serviços de saúde, bem como da possibilidade de garantir que seja produzido o necessário, no tempo e forma apropriados; pode ter perspectiva educativa, que abarca a participação ativa da equipe, por intermédio de ações/práticas educativas, além de ter o enfoque de articulação política relativo ao papel da supervisão como mediador entre a política institucional e a equipe, para viabilização do cuidado de enfermagem^(11)^.

A realização deste estudo é justificada pela peculiaridade do exercício da supervisão de enfermagem no SAMU, que requer agilidade nos processos decisórios e abordagem integral às necessidades dos pacientes^(11,12)^, somadas ao fato de que o enfermeiro e o técnico de enfermagem não estão no mesmo espaço físico durante a assistência^(7)^. Ademais, pesquisas científicas são essenciais para compreender a complexidade de aspectos organizacionais e processuais relativos à supervisão, cujos resultados possibilitem desenvolver estratégias para apoiar os enfermeiros, de forma a potencializar a qualificação dos cuidados prestados^(8,13)^. O último trabalho nacional sobre a especificidade da supervisão de enfermagem no SAMU foi publicado há uma década^(13)^. Nesse sentido, questiona-se: Quais fatores facilitam e quais dificultam a supervisão de enfermagem no SAMU? Diante do exposto, o estudo tem como objetivo analisar limitações e potencialidades da supervisão de enfermagem, segundo a equipe de enfermagem de um SAMU.

## MÉTODO

### Desenho do Estudo

Pesquisa descritiva, de abordagem qualitativa, utilizando a Técnica do Incidente Crítico (TIC), que requer o cumprimento dos seguintes passos: Determinação dos objetivos da atividade a ser executada; Elaboração das perguntas a serem feitas às pessoas que fornecerão os incidentes críticos da atividade a ser analisada; Delimitação da população; Coleta dos incidentes críticos; Análise do conteúdo dos incidentes coletados; Agrupamento e categorização dos incidentes críticos; Levantamento das frequências dos incidentes críticos positivos e negativos^(8,14)^.

Cabe destacar que o presente trabalho seguiu as recomendações do protocolo internacional *Consolidated Criteria for Reporting Qualitative Research* (COREQ)^(8,15)^ para apresentação dos resultados de pesquisa.

### Local do Estudo

A pesquisa foi realizada em base descentralizada de SAMU, em município do interior do estado de São Paulo, com população estimada de 127 mil habitantes, em 2022, distribuída em cerca de 400 km^2^ de território^(16)^. O serviço conta com duas USBs que atuam no atendimento dos munícipes e uma USA que atende à região e aos munícipes.

### População e Critérios de Seleção

Os participantes foram enfermeiros (ENF) e técnicos de enfermagem (TE) do SAMU, totalizando sete enfermeiros e quatorze técnicos de enfermagem, 21 participantes potenciais. Utilizou-se como critério de inclusão: profissionais atuantes, no serviço investigado, há no mínimo um ano, tempo que permite ao profissional ter experiência de inserção nas atividades da unidade.

### Coleta de Dados

Para coleta de dados foi organizado um roteiro de entrevista, de maneira a atender às recomendações da TIC^(8,14)^, composto por três tópicos: identificação do participante; duas questões abertas, com texto similar, um com referência positiva e outro com referência negativa, acerca de situações vividas ou observadas sobre a supervisão de enfermagem no SAMU, como as pessoas se comportaram e as consequências decorrentes da situação, além de outra questão aberta pedindo aos participantes sugestões para melhorar a supervisão de enfermagem no SAMU.

O roteiro passou por validação de face por juízes, enfermeiros-docentes, escolhidos intencionalmente em virtude do domínio do tema e do método e foi submetido a pré-teste, com profissionais que desempenham a mesma função dos participantes, porém em unidades distintas à da coleta, de modo a assegurar a qualidade e a aplicabilidade do roteiro da entrevista em condições reais. Ressalta-se que foram feitos ajustes de forma e não de conteúdo para a versão final do roteiro. A coleta de dados ocorreu no período de abril a junho de 2022, por intermédio de entrevistas semiestruturadas, presenciais e individuais, em um ambiente reservado, que tiveram duração aproximada de 30 minutos e foram conduzidas pela pesquisadora principal do estudo, após consentimento dos participantes.

### Análise e Tratamento dos Dados

As entrevistas foram gravadas em dois aparelhos digitais, transcritas na íntegra pela pesquisadora e conferidas com os respectivos áudios por outro pesquisador, sendo armazenadas em um banco de dados de um *e-mail* institucional do centro de pesquisa. Com o propósito de evitar a identificação dos participantes, as falas foram codificadas com “ENF” de enfermeiro ou “TE” de técnico de enfermagem, seguidas da letra “E” de Entrevista e posteriormente acrescidas pelos números (1, 2, 3.... até 17) na ordem sequencial em que foram realizadas, representando cada um dos participantes.

A pesquisadora principal sistematizou os dados e, considerando a análise da TIC, extraiu as situações, os comportamentos e as consequências, compondo os ICs, que receberam, a partir da visão dos participantes, referências positivas ou negativas, correspondendo às potencialidades e limitações da supervisão de enfermagem, respectivamente. Empregou-se, inicialmente, a estatística descritiva para quantificar os ICs e proceder à análise do conteúdo^(17)^.

Os dados foram analisados por meio da análise de conteúdo de Bardin^(18)^, submetidos a pré-análise, com leitura flutuante e preparo do material, incluindo correções gramaticais, excluídos vícios de linguagem, interferências e ruídos externos, visando permitir ao leitor a compreensão do sentido da entrevista; posteriormente, foi realizada a exploração do material com os relatos pertinentes a cada situação, comportamento e consequência, agrupados por similaridade de conteúdo; e finalmente, os resultados obtidos foram interpretados segundo o contexto do SAMU e pressupostos teóricos da supervisão de enfermagem.

### Aspectos Éticos

O estudo foi apreciado e aprovado pelo Comitê de Ética em Pesquisa da Escola de Enfermagem de Ribeirão Preto da Universidade de São Paulo, conforme parecer 4.240.326 (em 27 de agosto de 2020), de maneira a atender às determinações da Resolução nº 466/2012 do Conselho Nacional de Saúde. Todos os participantes assinaram o Termo de Consentimento Livre e Esclarecido, antes de realizar as entrevistas.

## RESULTADOS

Foram entrevistados 17 profissionais, distribuídos entre dez técnicos de enfermagem (59%) e sete enfermeiros (41%). Do total de 21 profissionais de enfermagem do SAMU, quatro não atenderam aos critérios de inclusão. A maioria dos entrevistados correspondeu ao sexo feminino (n = 10; 59%). Houve maior concentração de participantes na faixa etária de 41 a 50 anos (n = 8; 47%); entre 10 e 15 anos de formação profissional (n = 7; 41%), que atuavam na unidade entre seis e dez anos (n = 7; 41%).

Das 17 entrevistas, originaram-se 77 ICs, dos quais 17(22%) receberam referências positivas e 60(78%) negativas, agrupando-se esses ICs em quatro categorias: Singularidades da supervisão de enfermagem, com 34% de referências positivas e 66% referências negativas; Condições Organizativas, com 100% de referências negativas; Gestão de pessoas com 20% de referências positivas e 80% de referências negativas e Regulação de vagas, com 12% de referências positivas e 88% de referências negativas.

### Singularidades da Supervisão de Enfermagem

A categoria “Singularidades da supervisão de enfermagem” agrupa ICs relativos ao modo característico da supervisão de enfermagem no SAMU, no qual técnico e enfermeiro pertencem a equipes distintas. Houve predomínio de incidentes com referência negativa, como é possível constatar:

*Eu não sei o que o técnico está fazendo, a não ser que eu esteja com ele ou que seja um problema muito discrepante que eu ouço no rádio, mas eu não estou ouvindo rádio tempo todo; a supervisão aqui ela não é direta, ela é à distância, a enfermeira não tem contato com o técnico de enfermagem o tempo todo* (ENF E14).

*Às vezes, o técnico está fazendo alguma coisa inadequada; por exemplo, não estava fazendo massagem cardíaca corretamente, mas ele jamais iria me perguntar se eu não estivesse lá, acompanhando o atendimento.* (ENF E01)

*Aqui no SAMU a supervisão é muito indireta, a enfermeira não consegue estar junto com o técnico de enfermagem na ambulância o tempo todo, são viaturas separadas, a gente não está ali no momento exato do atendimento para supervisionar, orientar uma técnica, um cuidado*. (ENF E06).

Em contrapartida, são sugeridas formas para minimizar os aspectos negativos, com ICs que representam perspectivas positivas (34%):

*O que mais ajuda, quando tem problemas nos atendimentos, é poder esclarecer dúvidas com a supervisão de enfermagem, complementar, no sentido de agregar, entendeu? Mas, na maior parte do tempo, não fazem a supervisão direta.* (TE E7)

*Eu converso muito com a equipe sobre os atendimentos em que participo, me interesso pelo que eles realizam; quando tem alguma coisa diferente ou que eles discutiram, quero saber detalhes do que aconteceu; essa interação favorece a supervisão de enfermagem* (ENF E14)

*O hábito de chegar na base e discutir sobre o atendimento, tanto com a enfermeira, quanto entre nós, técnicos e socorristas, é sempre uma coisa positiva para refletir e aprender.* (TE E10)

No tocante à singularidade da supervisão de enfermagem, a ausência da supervisão direta revela-se um fator limitante, que decorre da especificidade do processo de trabalho em bases descentralizadas do SAMU.

### Condições Organizativas

A categoria “Condições Organizativas” agrupa incidentes que envolvem a limpeza e a conservação das unidades de atendimento móvel e da base do SAMU, gerenciamento e disponibilidade de materiais e insumos de trabalho.

*Quando tem problema, gente da USB fica com uma ambulância inferior, porque a USA não pode trabalhar com uma ambulância inferior, mas a básica pode, mas não é bom para o atendimento.* (TE E07)

*A limpeza, higiene, são complicadas aqui, a maioria da equipe não limpa e não vejo a supervisão atuando.* (TE E11)

*Eu assumi um plantão e não tive tempo de realizar o checklist da ambulância; saí para o atendimento e considerei que o colega tinha deixado tudo certo, era entrar e trabalhar. No local, era um paciente com BIPAP, que precisava estar ligado na rede de oxigênio para não dessaturar, e o torpedo de oxigênio estava vazio.* (TE E17)

*Eles nem sempre higienizam a ambulância, e eu não vejo a supervisão de enfermagem atuando continuamente para isso* (ENF E9)

Vale ressaltar que não houve aspectos positivos na categoria de incidentes “Condições Organizativas”.

### Gestão de Pessoas

A categoria “Gestão de Pessoas” agrupa incidentes referentes ao dimensionamento da equipe, manejo de escala e gerenciamento de conflitos. Apresentou maior frequência de incidentes negativos:

*A enfermeira não pode autorizar hora extra, mas precisa de troca de escala, tem muita Licença Saúde e precisa viabilizar o funcionamento do serviço.* (TE E3)

*Quando eu passei no concurso da prefeitura, fui informada que iria trabalhar no SAMU, que tinha vaga disponível, mas eu desejava trabalhar em outro tipo de serviço de saúde.* (TE E16)

*Eu já presenciei situações em que um funcionário queria agredir o outro, brigas devido à demora para responder à solicitação, conflitos que são gerados pelo tipo de personalidade de algumas pessoas.* (ENF E15)

No entanto, também foram evidenciados incidentes positivos:

*Na pandemia, a gente conseguiu ficar em dois enfermeiros no plantão; era possível sair nos atendimentos junto com o técnico, isso fazia uma boa diferença: poder ensinar, acompanhar o que e como está fazendo.* (ENF E6)

*A Covid, apesar de ter trazido tanta dor, trouxe algumas coisas boas de atualização de conhecimentos para a equipe de enfermagem.* (ENF E1)

### Regulação de Vagas

A categoria “Regulação de vagas” reuniu ICs referentes à relação dos profissionais de enfermagem e às demandas da Central de Regulação (CR) para o SAMU, com predomínio de incidentes negativos:

*A CR é fora do município, é regional e o pessoal da regulação não conhece muito bem a realidade local e isso complica o trabalho.* (ENF E1)

*Um tipo de discussão muito difícil que acontece é entre os tripulantes das unidades e o pessoal da CR; o enfermeiro fica nesse meio termo, de ter que orientar para não dar respostas grosseiras ou questionar o tipo de atendimento, mas as USBs sempre questionam e fica uma disputa com a CR.* (ENF E14)

*CR chama para atendimentos de baixíssima complexidade, eu diria atendimentos “banais”* (TE E12)

*Você está na viatura, se não for e acontecer alguma coisa com o paciente, você sabe qual é a minha posição! Vai, faz e, na hora em que chegar, relata o que está acontecendo; façam o melhor, usem o último recurso, tudo que puderem, e aí qualquer coisa depois a gente questiona e conversa com a CR.* (ENF E14)

## DISCUSSÃO

Em relação às variáveis sociodemográficas e profissionais dos participantes, verifica-se que os resultados são semelhantes aos achados de trabalho desenvolvido no mesmo contexto, na região metropolitana de São Paulo^(19)^. As competências são desenvolvidas ao longo dos anos de prática profissional, e o enfermeiro leva de dois a três anos para ter desempenho prático adequado, que geralmente decorre de aproximações sucessivas aos problemas cotidianos e possíveis soluções^(20)^.

Nos atendimentos básicos, o TE não está o tempo todo com o enfermeiro, o que torna a supervisão de enfermagem desafiadora, pois impossibilita a avaliação e o suporte da equipe de enfermagem no momento da assistência ao paciente. A supervisão de enfermagem deve ser conduzida como um processo dinâmico, a partir da análise situacional, seguida pela implementação de intervenções para alcançar os objetivos estabelecidos – que deverão ser reavaliados a partir dos resultados obtidos – e para realizar ajustes, se necessário for^(21)^.

A categoria “Singularidades da supervisão” teve predomínio de incidentes limitantes para o desempenho da supervisão de enfermagem. As características do processo de trabalho, no qual, para os atendimentos básicos, o TE não está o tempo todo com o enfermeiro, tornam a supervisão de enfermagem peculiar no SAMU. A superação de barreiras relativas à supervisão de enfermagem é multifatorial; existem intencionalidades e questões evidenciadas na prática cotidiana de trabalho^(10)^. O uso da supervisão sob uma abordagem educativa é potente, mas requer apoio institucional e investimento do enfermeiro no desenvolvimento de autonomia da equipe.

Os resultados evidenciam que os participantes indicaram caminhos que favoreceram a supervisão de enfermagem – com especial destaque às reuniões após os atendimentos – caminhos estes construídos de modo coletivo e horizontalizados, que repercutem positivamente no trabalho e favorecem a reflexão sobre a prática, oportunizam a troca de conhecimento e podem direcionar iniciativas que trazem um respaldo teórico mais robusto. Destaca-se a educação como ferramenta da supervisão de enfermagem, realizada a partir de *feedback* oportuno do enfermeiro para a equipe de TE e do coordenador de enfermagem para os enfermeiros. Um *feedback* claro e construtivo visa aprimorar a comunicação, aumentar a confiança e segurança no desempenho profissional^(8)^. A criação de espaços e oportunidades de escuta empática e utilização de medidas que proporcionem a interação dos membros da equipe são ações primordiais como possibilidade de qualificação da equipe e superação de desafios^(10)^.

Na categoria “Condições Organizativas” foram elencados somente incidentes negativos, ou seja, que limitam a supervisão de enfermagem. Cabe ressaltar que essa categoria agrupa incidentes que, comumente, transcendem o poder de deliberação do enfermeiro, uma vez que prover estrutura adequada, insumos e demais aspectos organizativos – para que os processos assistenciais e gerenciais aconteçam plenamente – é responsabilidade institucional. Entretanto, ainda assim, a responsabilidade por ações referentes à organização recai sobre esse profissional, o que corrobora seu caráter de referência no serviço.

Ressalta-se que infraestrutura, materiais e manutenções insuficientes são razões de insatisfação dos profissionais e fragilizam a assistência, assim como – somados à ausência de escala/organização para atividades inerentes à rotina, como limpeza, organização e reposição de materiais – fazem com que a equipe trabalhe de forma desarticulada e desestimulada, trazendo a falsa impressão de que se trata de atividades de menor importância, mas são essenciais para garantir o atendimento adequado e seguro de ocorrências. Inadequação e a insuficiência de materiais, de equipamentos e de veículos de suporte – aspectos relativos ao apoio institucional para o funcionamento do serviço – não estão na governabilidade do enfermeiro, mas repercutem em condições que podem representar riscos assistenciais e profissionais, trazendo sentimento de insegurança e desvalorização.

Ações como a manutenção dos veículos de suporte de vida são essenciais para um atendimento seguro e eficiente, uma vez que as unidades de suporte básico e avançado são o local de trabalho da equipe até o usuário chegar ao serviço de referência^(22)^. A inadequação de manutenção das unidades de suporte básico e avançado pode expor pacientes e a própria unidade de saúde a condições inseguras, que implicam à instituição sanções éticas e legais^(23)^.

A implementação de escalas para atividades cotidianas, como conferência de materiais, organização e limpeza, permite valorização da atividade pela equipe, de forma a favorecer a finalidade de controle da supervisão de enfermagem, no sentido de conferir padronização ao serviço. Ademais, o estresse ocupacional percebido por enfermeiros de emergência é agravado pela escassez de recursos, baixo suporte oferecido, má definição de papéis e atribuições, falta de autonomia e carga de trabalho excessiva^(24)^.

O desenvolvimento da supervisão de enfermagem também precisa considerar o contexto e as condições nas quais o trabalho da equipe de enfermagem e do enfermeiro está sendo desenvolvido; é pouco provável que se atinjam práticas de excelência em condições organizativas falhas; em cenários assim, muitas vezes, a supervisão de enfermagem fica reduzida a uma dimensão de controle de produção. O exercício da supervisão nos serviços beneficia a equipe com aumento do sentimento de apoio, favorece a redução da fadiga emocional, e melhora o relacionamento entre a equipe de enfermagem, o que pode refletir em melhora na satisfação do trabalho e no desenvolvimento das práticas profissionais^(25)^.

Na categoria “Gestão de pessoas”, houve predomínio de incidentes negativos. A dificuldade de comunicação e os conflitos presentes, tanto na equipe quanto entre órgãos de apoio, interferem no processo da prática da supervisão de enfermagem e causam impacto na satisfação profissional e no desempenho de atividades cotidianas. A falta de objetividade e clareza na comunicação pode resultar em planejamento inapropriado para o atendimento à ocorrência. Além disso, a falta de direcionamento específico da contratação de pessoal, para determinado serviço, pode implicar em profissionais insatisfeitos, por não conseguirem ser alocados nos locais de trabalho com os quais têm maior afinidade.

Nesse sentido, entende-se que a supervisão e a comunicação são saberes gerenciais primordiais para os enfermeiros, na condução da equipe, para o manejo de conflitos e para o alcance da amplitude da prática profissional no SAMU, exercendo a liderança de maneira democrática e horizontalizada, respaldados em conhecimentos técnicos e científicos^(26)^. Adversidades inerentes ao relacionamento interpessoal devem ser enfrentadas para além da busca de uma prática cotidiana humanizada, ética e colaborativa, e também para minimizar ou até evitar a legitimação de meios evasivos e defensivos frente a situações desafiadoras do cotidiano de trabalho^(10)^, de modo a favorecer a qualidade na assistência e preservar o bem-estar psicológico dos trabalhadores. A qualidade do ambiente e do trabalho é essencial, principalmente no cenário de pandemia^(27)^.

No contexto do SAMU, mudanças decorrentes da pandemia repercutiram em aumento do contingente de profissionais, um fator que potencializou a supervisão de enfermagem, viabilizou a oportunidade de experenciar desdobramentos positivos a partir da supervisão direta e do atendimento em conjunto com o TE. Porém, é preciso ressaltar que mudanças decorrentes da pandemia intensificaram os desafios já existentes na atuação do SAMU^(28)^.

O dimensionamento da equipe de enfermagem, aquém das necessidades do serviço, impacta na qualidade do atendimento nas diferentes áreas de atuação da enfermagem, gera sobrecarga de trabalho, favorece o estresse, a ocorrência de erros e de eventos adversos, fatores que comprometem a segurança do paciente^(29)^.

A promoção de ações educativas na perspectiva da educação continuada e permanente e a organização de ambientes seguros entre a equipe, para discussão de casos com fundamentação teórica da supervisão de enfermagem, em consonância com conhecimento técnico – assim como reuniões periódicas entre os enfermeiros, a fim de melhorar a prática profissional, trocar experiências acerca de questões gerenciais e organizacionais, estabelecendo ambientes para a construção coletiva, incentivando a força interna da equipe – foram consideradas fatores potencializadores da supervisão de enfermagem nesse contexto de trabalho. Um ambiente de apoio entre os profissionais para trocas de experiências, comunicação, promoção de treinamentos sobre temas pertinentes ao contexto é uma contribuição positiva para a equipe como um todo e, por consequência, uma melhoria da qualificação do cuidado. Esse apoio inclui protocolos e técnicas assistenciais, limpeza, uso de equipamentos e tecnologias ligadas ao cuidado direto, assim como discussões de casos após as ocorrências, já presentes no serviço, que consideram as reflexões sobre situações vivenciadas.

O apoio à inclusão de atividades educativas (cursos e treinamentos) – realizado em horário de trabalho, sem prejuízo para o trabalhador e com dimensionamento adequado do quadro de profissionais para cobertura da escala de trabalho – poderia ser uma forma de enfrentamento dos desafios inerentes à prática da supervisão de enfermagem no SAMU. A partir da análise das oportunidades de aprimoramento, torna-se possível impulsionar o desenvolvimento de competências dos profissionais por meio da aplicação prática do conhecimento^(30)^.

A categoria “Regulação de vagas” concentrou maior contingente de referências negativas, o que representa limitações para a supervisão de enfermagem. Os incidentes referentes à central de regulação de vagas podem ser entendidos como complexos, uma vez que – embora sejam interface crucial entre sistema e serviços de saúde com usuários e profissionais – muitas vezes, desencadeiam ruídos de comunicação e conflitos, que repercutem na satisfação e adequação do atendimento ao usuário, nas atribuições e responsabilidades de diferentes pontos da rede de atenção e na atuação das respectivas equipes de saúde. Particularmente, para a enfermagem, pode-se inferir que representa a dimensão da articulação política da supervisão.

Os profissionais do SAMU dependem diretamente da orientação e do bom funcionamento da Central de Regulação de Urgência/Emergência (CRUE) para a realização do seu trabalho; por outro lado, não possuem a visibilidade sobre a disponibilidade de vagas e todo o contexto de saúde da região, no momento do atendimento. Sendo assim, é necessário que se estabeleça uma relação de confiança, com o propósito de promover o atendimento ágil, seguro e eficiente ao paciente, minimizando os agravos à saúde.

Alguns usuários do SAMU desconhecem ou não foram esclarecidos sobre o serviço de saúde; estes devem buscar a razão pela qual optam pelo atendimento que julgam apropriado para o problema enfrentado naquele momento. É comum o uso desse serviço como meio de transporte, para solução de demandas sociais, ou como uma porta de entrada ao SUS, fora da situação de urgência, por ser um serviço em que o usuário encontrará, de forma rápida, uma equipe completa de profissionais, tratamento imediato e serviços de maior complexidade^(22)^.

O exposto evidencia aspectos macro-organizacionais, que repercutem também na supervisão de enfermagem; por exemplo, ruídos na interface com a CRUE, que podem causar insatisfação nos profissionais, resultar em conflitos e atrasos no atendimento, condições que independem da atuação direta dos profissionais do SAMU. Nesse sentido, as estratégias de enfrentamento não podem ser simplistas; entretanto, destacam-se, de modo oportuno, a capacidade de usar estratégias educativas tanto junto à equipe de enfermagem para mitigar problemas de comunicação e conflitos, quanto a oportunidade de a equipe esclarecer a população sobre uso do SAMU e outros serviços de saúde.

Como limitação do estudo, ressalta-se a análise de apenas um serviço, que restringe a possibilidade de análise ampliada do objeto de estudo; ademais, à época, estava começando a flexibilização das medidas de segurança relativas à pandemia de Covid-19 e estava em curso um processo de readequação dos serviços de saúde. Entretanto, apesar das limitações, o estudo contribui significativamente para a área da enfermagem, ao destacar aspectos que limitam e potencializam a supervisão de enfermagem, oferecendo *insights* valiosos para a melhoria da prática profissional e a criação de um ambiente de aprendizado e diálogo. Finalizando, não se pretende propor um guia ou modelo de trabalho, mas os resultados possibilitam sugerir aspectos a serem considerados no enfoque referente à supervisão de enfermagem no SAMU, como a possibilidade de explorar a prática da supervisão direta sempre que possível, com o enfermeiro como membro da equipe de suporte básico junto ao técnico de enfermagem.

A partir dos resultados e discussão, apresenta-se a síntese de aspectos que potencializam a realização da supervisão de enfermagem no SAMU: o apoio institucional no que tange a aspectos organizativos como veículo de transporte, materiais de limpeza e manutenções e no que se refere a instrumentos de ajustes para suprir demandas emergenciais de escala, como hora extra; o apoio à inclusão de atividades educativas em horário de trabalho, sem prejuízo para o trabalhador, realizando o uso da educação como ferramenta da supervisão de enfermagem, a partir de “*feedback”* oportuno; o encorajamento de ambiente de apoio entre os profissionais, visando estimular trocas de experiências; o fomento às discussões de casos após as ocorrências, de modo a considerar reflexões sobre situações vivenciadas, analisadas e pautadas no conhecimento técnico-científico e ético; reuniões periódicas com a equipe, como oportunidades de troca de conhecimento, com comunicação clara e objetiva, horizontalizada, capaz de refletir na valorização dos profissionais e suas funções; dimensionamento adequado do quadro de profissionais para cobertura da escala de trabalho junto da organização do serviço para atividades cotidianas, a partir de ferramentas, como escalas de trabalho, protocolos e procedimentos operacionais de rotinas de limpeza, checagem e reposição de materiais; estímulo e realização da supervisão direta do enfermeiro durante o atendimento da equipe de SBV, sempre que possível, com o objetivo de apoio, avaliação da prática profissional e realização de intervenções necessárias durante a prática ou por meio de medidas educativas após o atendimento.

Ainda considerando-se os resultados e discussão, apresenta-se a síntese de fatores limitantes para o desempenho da supervisão de enfermagem no SAMU: O próprio processo de trabalho direciona a equipe a partir do tipo de atendimento, o que repercute em fragmentação da equipe, uma vez que, nos atendimentos da equipe de Suporte Básico de Vida (SBV), o TE e o enfermeiro não ocupam o mesmo espaço, o que torna a supervisão de enfermagem desafiadora nesse serviço, visto que não há possibilidade de avaliação e suporte da equipe de enfermagem no momento da assistência ao paciente. Quanto aos aspectos organizativos, a inadequação e insuficiência de materiais, de manutenção dos equipamentos e de veículos de suporte, não estão na governabilidade do enfermeiro, mas repercutem em condições que podem representar riscos assistenciais e profissionais; ainda nessa temática, a ausência de organização para atividades inerentes à rotina, como limpeza, organização e reposição de materiais, faz com que a equipe trabalhe de forma desarticulada e desestimulada e impacta no atendimento adequado e seguro das ocorrências. Somam-se a esses fatores as dificuldades de comunicação e conflitos presentes tanto na equipe quanto entre órgãos de apoio.

## CONCLUSÃO

Com base nos resultados alcançados nesta pesquisa, tornou-se possível a identificação do predomínio de aspectos que limitam o desempenho da supervisão de enfermagem no contexto do SAMU. Isso destaca a premência de um olhar mais atento – particularmente no que diz respeito à supervisão de enfermagem – aos requisitos críticos necessários para a execução dessa atividade e à insuficiente abordagem educacional dedicada a esse componente gerencial, no contexto estudado.

Os resultados estimulam a reflexão sobre o desempenho do papel do enfermeiro no exercício da supervisão, mas também revelam aspectos relevantes relacionados ao gerenciamento do cuidado no SAMU. Esse cenário contribui significativamente para identificar e aprimorar pontos críticos, fornecendo apoio ao enfermeiro, no que se refere à prática da gestão do serviço, gerenciamento da equipe e realização da assistência. Nesse contexto, mesmo que os resultados não sejam passíveis de generalização, é plausível que outras unidades em contextos semelhantes possam extrair benefícios a partir das investigações apresentadas neste estudo.

Há muito que avançar nos conhecimentos teórico-práticos acerca do exercício da supervisão de enfermagem no SAMU, em especial sob a perspectiva educativa, que, mesmo apontada como um recurso utilizado, ainda se apresenta limitada em sua potencialidade.

## Data Availability

O conjunto de dados que dá suporte aos resultados deste estudo não está disponível publicamente.

# Translation: Supervisión de enfermería en el servicio móvil de atención de urgencias prehospitalarias

**DOI:** 10.1590/1980-220X-REEUSP-2024-0238es

**Authors:** Camila Galiano, André Almeida de Moura, Josué Souza Gleriano, Vivian Aline Mininel, Mayra de Cássia Trovó, Mariana Fraga de Figueiredo, Bethania Ferreira Goulart, Lucieli Dias Pedreschi Chaves

**Affiliations:** 1 Universidade de São Paulo, Escola de Enfermagem de Ribeirão Preto, Ribeirão Preto, SP, Brasil.; 2 Universidade de São Paulo, Escola de Enfermagem, Departamento de Orientação Profissional, São Paulo, SP, Brasil.; 3 Universidade do Estado de Mato Grosso, Enfermagem, Tangará da Serra, MT, Brasil.; 4 Universidade Federal de São Carlos, Enfermagem, São Carlos, SP, Brasil.; 5 Universidade Federal do Triângulo Mineiro, Enfermagem, Uberaba, MG, Brasil.

**Keywords:** Supervisión de Enfermería, Enfermería, Servicios Médicos de Urgência

## Abstract

**Objetivo:**

Analizar las limitaciones y el potencial de la supervisión de enfermería, según el equipo de enfermería, de un Servicio de Atención Médica de Urgencias.

**Método:**

Investigación descriptiva, de enfoque cualitativo, utilizando la técnica del incidente crítico. Participaron enfermeros y técnicos de enfermería. Los datos se recopilaron mediante entrevistas semiestructuradas, individuales, grabadas y posteriormente transcritas, seguidas de la agrupación y categorización de los Incidentes Críticos, utilizando el análisis de contenido de Bardin.

**Resultados:**

De las entrevistas surgieron 77 Incidentes Críticos, el 22% recibieron referencias positivas y el 78% negativas, lo que indica un predominio de factores que limitan la supervisión. Estos factores se clasificaron en «Singularidades de la supervisión de enfermería», «Condiciones organizativas», «Gestión de personas» y «Regulación de plazas».

**Conclusión:**

Factores potenciadores: apoyo institucional, educación como herramienta de supervisión, reuniones de equipo, «*feedback»* oportuno y gestión participativa; factores limitantes: supervisión indirecta de enfermería (enfermeros y técnicos en equipos distintos), falta de materiales y de mantenimiento y apoyo institucional, sobrecarga de trabajo del enfermero, conflictos y falta de comunicación.

## INTRODUCCIÓN

El Servicio de Atención Médica de Urgencias (SAMU) es un servicio público complejo que se presta en el contexto de la Red de Atención a Urgencias y Emergencias (RUE); entre otras actividades, se caracteriza se caracteriza por centrarse en situaciones de urgencia y emergencia prehospitalarias, con la atención y el derivación de los usuarios para la continuidad de la asistencia, además de realizar el transporte de pacientes críticos entre unidades de salud^(1)^. La atención prehospitalaria en yhgBrasil, hasta la década de 1990, seguía el modelo estadounidense de Atención Prehospitalaria (APH), con variaciones en su organización en los diferentes estados; a partir de 1997, las normativas del Ministerio de Salud (MS), influenciadas por el modelo francés, consolidaron la atención móvil de urgencia; y el decreto MS 2048, de 2022, redefinió el sistema brasileño de atención a urgencias y emergencias, mezclando los modelos estadounidense y francés, consolidando el SAMU^(2)^.

El SAMU utiliza diferentes tipos de unidades móviles para atender las emergencias, pero esencialmente el modelo terrestre se compone de la Unidad de Soporte Básico (USB) de vida, tripulada por el conductor del vehículo de urgencia y un técnico o auxiliar de enfermería, y la Unidad de Soporte Avanzado (USA) de vida, tripulada por un conductor de vehículo de urgencia, un enfermero y un médico^(3)^. Se trata de una estrategia eficaz de primeros auxilios, estabilización y traslado de pacientes a los servicios de referencia. Cabe destacar que la enfermería desempeña un papel relevante en este servicio, cuya constante evolución exige profesionales cualificados, capaces de atender las necesidades sanitarias en la atención prehospitalaria de urgencia, así como en el transporte hacia y entre hospitales, con énfasis en la promoción, la protección y la restauración de la salud, además de desarrollar acciones para garantizar el buen clima organizacional y la satisfacción del equipo en el trabajo^(4)^.

El enfermero desempeña un papel central, tanto en la asistencia directa al paciente como en la coordinación de los equipos, y su actuación profesional está regulada por legislaciones específicas, como la Resolución del Consejo Federal de Enfermería (COFEN) n.º 713/2023, que actualiza las normas de actuación en el APH móvil, y el Dictamen del Consejo Regional de Enfermería de São Paulo (COREN-SP) n.º 005/2019, que define el perfil y las competencias del técnico de enfermería en este ámbito. Estas leyes refuerzan la importancia del enfermero en la gestión de recursos, la toma de decisiones y la regulación de las urgencias, esenciales para la eficacia de la atención prehospitalaria, en consonancia con las directrices establecidas por el SAMU para garantizar la calidad y la seguridad en la atención al paciente^(5,6,7)^.

En la coordinación del proceso asistencial, la supervisión de enfermería identifica necesidades asistenciales y gerenciales específicas, para establecer planes y metas que contribuyan a un enfoque integral del paciente^(8)^. Cabe destacar que el enfermero tiene la atribución exclusiva de supervisar al equipo de enfermería, en un proceso educativo de integración y coordinación, que da como resultado una atención cualificada de las necesidades de los pacientes y las instituciones^(9)^.

La supervisión de enfermería puede entenderse desde diferentes perspectivas, como un instrumento de gestión dinámico, que desde hace tiempo se enfrenta al reto de avanzar en formas de desempeño que garanticen la producción adecuada de acciones asistenciales, desde una perspectiva integral y oportuna, pero con un enfoque más participativo, colaborativo e intergeneracional^(10)^. La supervisión puede centrarse en el control de la organización del trabajo, de la estandarización interna de los servicios de salud, así como de la posibilidad de garantizar que se produzca lo necesario, en el momento y de la forma adecuados; puede tener una perspectiva educativa, que abarca la participación activa del equipo, a través de acciones/prácticas educativas, además de tener un enfoque de articulación política relativo al papel de la supervisión como mediador entre la política institucional y el equipo, para viabilizar la atención de enfermería^(11)^.

La realización de este estudio se justifica por la peculiaridad del ejercicio de la supervisión de enfermería en el SAMU, que requiere agilidad en los procesos de toma de decisiones y un enfoque integral de las necesidades de los pacientes^(11,12)^, además del hecho de que el enfermero y el técnico de enfermería no se encuentran en el mismo espacio físico durante la asistencia^(7)^. Además, las investigaciones científicas son esenciales para comprender la complejidad de los aspectos organizativos y procedimentales relacionados con la supervisión, cuyos resultados permiten desarrollar estrategias para apoyar a los enfermeros, con el fin de potenciar la cualificación de los cuidados prestados^(8,13)^. El último trabajo nacional sobre la especificidad de la supervisión de enfermería en el SAMU se publicó hace una década^(13)^. En este sentido, se plantea la siguiente pregunta: ¿Qué factores facilitan y cuáles dificultan la supervisión de enfermería en el SAMU? Ante lo expuesto, el estudio tiene como objetivo analizar las limitaciones y el potencial de la supervisión de enfermería, según el equipo de enfermería de un SAMU.

## MÉTODO

### Diseño del Estudio

Investigación descriptiva, de enfoque cualitativo, utilizando la Técnica del Incidente Crítico (TIC), que requiere el cumplimiento de los siguientes pasos: Determinación de los objetivos de la actividad a realizar; Elaboración de las preguntas que se harán a las personas que proporcionarán los incidentes críticos de la actividad a analizar; Delimitación de la población; Recolección de los incidentes críticos; Análisis del contenido de los incidentes recolectados; Agrupación y categorización de los incidentes críticos; Levantamiento de las frecuencias de los incidentes críticos positivos y negativos^(8,14)^.

Cabe destacar que el presente trabajo siguió las recomendaci­ones del protocolo internacional *Criterios consolidados para la presentación de informes de investigación cualitativa* (COREQ)^(8,15)^ para la presentación de los resultados de la investigación.

### Lugar del Estudio

La investigación se llevó a cabo de forma descentralizada en el SAMU, en un municipio del interior del estado de São Paulo, con una población estimada de 127 000 habitantes en 2022, distribuidos en unos 400 km^2^ de territorio^(16)^. El servicio cuenta con dos USB que atienden a los habitantes del municipio y una USA que atiende a la región y a los habitantes.

### Población y Criterios de Selección

Los participantes fueron enfermeros/as (ENF) y técnicos/as de enfermería (TE) del SAMU, con un total de siete enfermeros y catorce técnicos de enfermería, 21 participantes potenciales. Se utilizó como criterio de inclusión: profesionales en activo en el servicio investigado desde hace al menos un año, tiempo que permite al profesional tener experiencia en las actividades de la unidad.

### Recolección de Datos

Para la recolección de datos se organizó un guion de entrevista, de manera que se cumplieran las recomendaciones de la TIC^(8,14)^, compuesto por tres temas: identificación del participante; dos preguntas abiertas, con texto similar, una con referencia positiva y otra con referencia negativa, sobre situaciones vividas u observadas sobre la supervisión de enfermería en el SAMU, cómo se comportaron las personas y las consecuencias derivadas de la situación, además de otra pregunta abierta en la que se pedía a los participantes sugerencias para mejorar la supervisión de enfermería en el SAMU.

El guion fue validado por jueces, enfermeros docentes, elegidos intencionalmente por su dominio del tema y del método, y fue sometido a una prueba previa con profesionales que desempeñan la misma función que los participantes, pero en unidades distintas a las de la recolección, con el fin de garantizar la calidad y la aplicabilidad del guion de la entrevista en condiciones reales. Cabe destacar que se realizaron ajustes de forma, pero no de contenido, para la versión final del guion. La recolección de datos se realizó entre abril y junio de 2022, mediante entrevistas semiestructuradas, presenciales e individuales, en un entorno reservado, con una duración aproximada de 30 minutos y fueron realizadas por la investigadora principal del estudio, tras el consentimiento de los participantes.

### Análisis y Tratamiento de los Datos

Las entrevistas se grabaron en dos dispositivos digitales, fueron transcritas íntegramente por la investigadora y verificadas con los respectivos audios por otro investigador, y se almacenaron en una base de datos de un correo electrónico institucional del centro de investigación. Con el fin de evitar la identificación de los participantes, las declaraciones se codificaron con «ENF» para enfermero/a o «TE» para técnico/a de enfermería, seguidas de la letra «E» de entrevista y posteriormente se añadieron los números (1, 2, 3... hasta 17) en el orden secuencial en que se realizaron, representando a cada uno de los participantes.

La investigadora principal sistematizó los datos y, teniendo en cuenta el análisis de las TIC, extrajo las situaciones, los comportamientos y las consecuencias, componiendo los IC, que recibieron, desde el punto de vista de los participantes, referencias positivas o negativas, correspondientes a las potencialidades y limitaciones de la supervisión de enfermería, respectivamente. Inicialmente, se utilizó la estadística descriptiva para cuantificar los IC y proceder al análisis del contenido^(17)^.

Los datos fueron analizados mediante el análisis de contenido de Bardin^(18)^, sometidos a un preanálisis, con lectura flotante y preparación del material, incluyendo correcciones gramaticales, excluyendo vicios del lenguaje, interferencias y ruidos externos, con el fin de permitir al lector comprender el sentido de la entrevista; posteriormente, se llevó a cabo la exploración del material con los relatos pertinentes a cada situación, comportamiento y consecuencia, agrupados por similitud de contenido; y, finalmente, los resultados obtenidos se interpretaron según el contexto del SAMU y los supuestos teóricos de la supervisión de enfermería.

### Aspectos Éticos

El estudio fue evaluado y aprobado por el Comité de Ética en Investigación de la Escuela de Enfermería de Ribeirão Preto de la Universidad de São Paulo, según el dictamen 4.240.326 (de 27 de agosto de 2020), a fin de cumplir con las disposiciones de la Resolución n.º 466/2012 del Consejo Nacional de Salud. Todos los participantes firmaron el Término de Consentimiento Libre y Esclarecido antes de realizar las entrevistas.

## RESULTADOS

Se entrevistó a 17 profesionales, distribuidos entre diez técnicos de enfermería (59%) y siete enfermeros (41%). Del total de 21 profesionales de enfermería del SAMU, cuatro no cumplieron los criterios de inclusión. La mayoría de los entrevistados eran mujeres (n = 10; 59%). Hubo una mayor concentración de participantes en el rango de edad de 41 a 50 años (n = 8; 47%); entre 10 y 15 años de formación profesional (n = 7; 41%), que trabajaban en la unidad entre seis y diez años (n = 7; 41%).

De las 17 entrevistas, se obtuvieron 77 IC, de los cuales 17(22%) recibieron referencias positivas y 60(78%) negativas, agrupándose estos IC en cuatro categorías: Singularidades de la supervisión de enfermería, con un 34% de referencias positivas y un 66% de referencias negativas; Condiciones organizativas, con un 100% de referencias negativas; Gestión de personas, con un 20% de referencias positivas y un 80% de referencias negativas, y Regulación de plazas, con un 12% de referencias positivas y un 88% de referencias negativas.

### Singularidades de la Supervisión de Enfermería

La categoría «Singularidades de la supervisión de enfermería» agrupa los IC relacionados con el modo característico de la supervisión de enfermería en el SAMU, en el que los técnicos y los enfermeros pertenecen a equipos distintos. Predominaron los incidentes con referencia negativa, como se puede observar:

*No sé lo que está haciendo el técnico, a menos que esté con él o que sea un problema muy grave que oigo por la radio, pero no estoy escuchando la radio todo el tiempo; la supervisión aquí no es directa, es a distancia, la enfermera no tiene contacto con el técnico de enfermería todo el tiempo* (ENF E14).

*A veces, el técnico está haciendo algo inadecuado; por ejemplo, no estaba haciendo el masaje cardíaco correctamente, pero nunca me lo habría preguntado si yo no estuviera allí, supervisando la atención.* (ENF E01)

*Aquí en el SAMU la supervisión es muy indirecta, la enfermera no puede estar con el técnico de enfermería en la ambulancia todo el tiempo, son vehículos separados, no estamos allí en el momento exacto de la atención para supervisar, orientar a un técnico, un cuidado*. (ENF E06).

Por otro lado, se sugieren formas de minimizar los aspectos negativos, con IC que representan perspectivas positivas (34%):

*Lo que más ayuda, cuando hay problemas en la atención, es poder aclarar dudas con la supervisión de enfermería, complementar, en el sentido de agregar, ¿entiendes? Pero, la mayor parte del tiempo, no hacen supervisión directa.* (TE E7)

*Hablo mucho con el equipo sobre las atenciones en las que participo, me interesa lo que hacen; cuando hay algo diferente o que han discutido, quiero saber los detalles de lo que ha pasado; esta interacción favorece la supervisión de enfermería* (ENF E14)

*La costumbre de llegar a la base y discutir sobre la atención, tanto con la enfermera como entre nosotros, técnicos y socorristas, siempre es algo positivo para reflexionar y aprender.* (TE E10)

En lo que respecta a la singularidad de la supervisión de enfermería, la ausencia de supervisión directa se revela como un factor limitante, que se deriva de la especificidad del proceso de trabajo en las bases descentralizadas del SAMU.

### Condiciones Organizativas

La categoría «Condiciones organizativas» agrupa los incidentes relacionados con la limpieza y el mantenimiento de las unidades de atención móvil y de la base del SAMU, la gestión y la disponibilidad de materiales y suministros de trabajo.

*Cuando hay un problema, el personal de la UBS se queda con una ambulancia inferior, porque la USA no puede trabajar con una ambulancia inferior, pero la básica sí puede, aunque no es buena para la atención.* (TE E07)

*La limpieza y la higiene son complicadas aquí, la mayoría del personal no limpia y no veo que haya supervisión.* (TE E11)

*Asumí un turno y no tuve tiempo de realizar la lista de comprobación de la ambulancia; salí para atender y consideré que mi compañero lo había dejado todo en orden, solo tenía que entrar y trabajar. En el lugar, había un paciente con BIPAP, que necesitaba estar conectado a la red de oxígeno para no desaturarse, y el torpedo de oxígeno estaba vacío.* (TE E17)

*No siempre desinfectan la ambulancia y no veo que la supervisión de enfermería actúe continuamente para ello*. (ENF E9)

Cabe destacar que no hubo aspectos positivos en la categoría de incidentes «Condiciones organizativas».

### Gestión de Personas

La categoría «Gestión de personas» agrupa los incidentes relacionados con la dotación de personal, la gestión de turnos y la gestión de conflictos. Presentó una mayor frecuencia de incidentes negativos:

*La enfermera no puede autorizar horas extras, pero necesita un cambio de turno, hay muchas bajas por enfermedad y hay que garantizar el funcionamiento del servicio.* (TE E3)

*Cuando aprobé el concurso de la alcaldía, me informaron que iba a trabajar en el SAMU, que había una vacante disponible, pero yo quería trabajar en otro tipo de servicio de salud.* (TE E16)

*Ya he presenciado situaciones en las que un empleado quería agredir a otro, peleas por demoras en responder a una solicitud, conflictos generados por el tipo de personalidad de algunas personas.* (ENF E15)

Sin embargo, también se evidenciaron incidentes positivos:

*Durante la pandemia, conseguimos quedarnos con dos enfermeros de guardia; era posible salir a atender junto con el técnico, lo que marcaba una gran diferencia: poder enseñar, acompañar lo que se está haciendo y cómo se está haciendo.* (ENF E6)

*El Covid, a pesar de haber traído tanto dolor, trajo algunas cosas buenas, como la actualización de conocimientos para el equipo de enfermería.* (ENF E1)

### Regulación de Plazas

La categoría «Regulación de plazas» reunió IC relacionados con la relación entre los profesionales de enfermería y las demandas de la Central de Regulación (CR) para el SAMU, con predominio de incidentes negativos:

*La CR está fuera del municipio, es regional y el personal de regulación no conoce muy bien la realidad local, lo que complica el trabajo.* (ENF E1)

*Un tipo de discusión muy difícil que se produce es entre los tripulantes de las unidades y el personal de la CR; el enfermero se queda en medio, teniendo que orientar para no dar respuestas groseras o cuestionar el tipo de atención, pero las USB siempre cuestionan y se produce una disputa con la CR.* (ENF E14)

*La CR llama para atender casos de muy baja complejidad, yo diría que son casos «banales»*. (TE E12)

*Estás en la ambulancia, si no vas y le pasa algo al paciente, ¡ya sabes cuál es mi postura! Ve, hazlo y, cuando llegues, informa de lo que está pasando; hagan lo mejor, utilicen el último recurso, todo lo que puedas, y luego ya preguntamos y hablamos con la CR.* (ENF E14)

## DISCUSIÓN

En relación con las variables sociodemográficas y profesionales de los participantes, se observa que los resultados son similares a los hallados en trabajos desarrollados en el mismo contexto, en la región metropolitana de São Paulo^(19)^. Las competencias se desarrollan a lo largo de los años de práctica profesional, y el enfermero tarda entre dos y tres años en alcanzar un rendimiento práctico adecuado, que generalmente se deriva de sucesivos acercamientos a los problemas cotidianos y a las posibles soluciones^(20)^.

En la atención básica, el/la TE no está todo el tiempo con el enfermero, lo que dificulta la supervisión de la enfermería, ya que imposibilita la evaluación y el apoyo del equipo de enfermería en el momento de la atención al paciente. La supervisión de enfermería debe llevarse a cabo como un proceso dinámico, a partir del análisis de la situación, seguido de la implementación de intervenciones para alcanzar los objetivos establecidos, que deberán reevaluarse a partir de los resultados obtenidos, y para realizar ajustes, si fuera necesario^(21)^.

La categoría «Singularidades de la supervisión» presentó una predominancia de incidentes que limitaban el desempeño de la supervisión de enfermería. Las características del proceso de trabajo, en el que, para la atención básica, el/la TE no está todo el tiempo con el enfermero, hacen que la supervisión de enfermería sea peculiar en el SAMU. La superación de las barreras relacionadas con la supervisión de enfermería es multifactorial; existen intencionalidades y cuestiones que se evidencian en la práctica cotidiana del trabajo^(10)^. El uso de la supervisión desde un enfoque educativo es potente, pero requiere apoyo institucional y la inversión del enfermero en el desarrollo de la autonomía del equipo.

Los resultados evidencian que los participantes indicaron vías que favorecieron la supervisión de enfermería, con especial énfasis en las reuniones posteriores a las atenciones, vías estas construidas de manera colectiva y horizontalizada, que repercuten positivamente en el trabajo y favorecen la reflexión sobre la práctica, facilitan el intercambio de conocimientos y pueden orientar iniciativas que aportan un respaldo teórico más sólido. Cabe destacar la educación como herramienta de supervisión de enfermería, llevada a cabo a partir de *feedback* oportuno del enfermero al equipo de TE y del coordinador de enfermería a los enfermeros. Un *feedback* claro y constructivo tiene como objetivo mejorar la comunicación, aumentar la confianza y la seguridad en el desempeño profesional^(8)^. La creación de espacios y oportunidades para la escucha empática y el uso de medidas que propicien la interacción entre los miembros del equipo son acciones fundamentales para la cualificación del equipo y la superación de retos^(10)^.

En la categoría «Condiciones organizativas» solo se enumeraron incidentes negativos, es decir, que limitan la supervisión de enfermería. Cabe destacar que esta categoría agrupa incidentes que, por lo general, trascienden el poder de deliberación del enfermero, ya que proporcionar una estructura adecuada, insumos y otros aspectos organizativos —para que los procesos asistenciales y gerenciales se desarrollen plenamente— es responsabilidad institucional. Sin embargo, la responsabilidad de las acciones relacionadas con la organización recae en este profesional, lo que corrobora su carácter de referencia en el servicio.

Cabe destacar que la infraestructura, los materiales y el mantenimiento insuficientes son motivos de insatisfacción de los profesionales y debilitan la asistencia, así como, sumados a la falta de escala/organización para las actividades inherentes a la rutina, como la limpieza, organización y reposición de materiales, hacen que el equipo trabaje de forma descoordinada y desmotivada, dando la falsa impresión de que se trata de actividades de menor importancia, pero que son esenciales para garantizar una atención adecuada y segura de las incidencias. La inadecuación e insuficiencia de materiales, equipos y vehículos de apoyo —aspectos relacionados con el apoyo institucional para el funcionamiento del servicio— no dependen de la gobernanza del enfermero, pero repercuten en condiciones que pueden suponer riesgos asistenciales y profesionales, generando un sentimiento de inseguridad y desvalorización.

Acciones como el mantenimiento de los vehículos de soporte vital son esenciales para una atención segura y eficiente, ya que las unidades de soporte básico y avanzado son el lugar de trabajo del equipo hasta que el usuario llega al servicio de referencia^(22)^. El mantenimiento inadecuado de las unidades de soporte básico y avanzado puede exponer a los pacientes y a la propia unidad de salud a condiciones inseguras, lo que implica sanciones éticas y legales para la institución^(23)^.

La implementación de turnos para las actividades cotidianas, como la revisión de materiales, la organización y la limpieza, permite que el equipo valore la actividad, lo que favorece el objetivo de control de la supervisión de enfermería, en el sentido de estandarizar el servicio. Además, el estrés laboral percibido por los enfermeros de urgencias se ve agravado por la escasez de recursos, el escaso apoyo ofrecido, la mala definición de funciones y atribuciones, la falta de autonomía y la carga de trabajo excesiva^(24)^.

El desarrollo de la supervisión de enfermería también debe tener en cuenta el contexto y las condiciones en las que se desarrolla el trabajo del equipo de enfermería y del enfermero; es poco probable que se alcancen prácticas de excelencia en condiciones organizativas deficientes; en escenarios como estos, a menudo, la supervisión de enfermería se reduce a una dimensión de control de la producción. El ejercicio de la supervisión en los servicios beneficia al equipo con un aumento del sentimiento de apoyo, favorece la reducción de la fatiga emocional y mejora la relación entre el equipo de enfermería, lo que puede reflejarse en una mejora de la satisfacción laboral y en el desarrollo de las prácticas profesionales^(25)^.

En la categoría «Gestión de personas», predominaron los incidentes negativos. La dificultad de comunicación y los conflictos presentes, tanto en el equipo como entre los órganos de apoyo, interfieren en el proceso de la práctica de la supervisión de enfermería y repercuten en la satisfacción profesional y en el desempeño de las actividades cotidianas. La falta de objetividad y claridad en la comunicación puede dar lugar a una planificación inadecuada para atender la incidencia. Además, la falta de orientación específica en la contratación de personal para un servicio determinado puede dar lugar a profesionales insatisfechos, al no poder ser asignados a los lugares de trabajo con los que tienen mayor afinidad.

En este sentido, se entiende que la supervisión y la comunicación son conocimientos gerenciales fundamentales para los enfermeros en la dirección del equipo, la gestión de conflictos y el alcance de la amplitud de la práctica profesional en el SAMU, ejerciendo el liderazgo de manera democrática y horizontal, respaldados por conocimientos técnicos y científicos^(26)^. Las adversidades inherentes a las relaciones interpersonales deben afrontarse más allá de la búsqueda de una práctica cotidiana humanizada, ética y colaborativa, y también para minimizar o incluso evitar la legitimación de medios evasivos y defensivos ante situaciones desafiantes del día a día del trabajo^(10)^, con el fin de favorecer la calidad de la asistencia y preservar el bienestar psicológico de los trabajadores. La calidad del entorno y del trabajo es esencial, especialmente en el contexto de una pandemia^(27)^.

En el contexto del SAMU, los cambios derivados de la pandemia se han traducido en un aumento del número de profesionales, lo que ha potenciado la supervisión de la enfermería y ha brindado la oportunidad de experimentar desarrollos positivos a partir de la supervisión directa y la atención conjunta con el/la TE. Sin embargo, cabe destacar que los cambios derivados de la pandemia han intensificado los retos ya existentes en la actuación del SAMU^(28)^.

La dotación de personal de enfermería, inferior a las necesidades del servicio, repercute en la calidad de la atención en las diferentes áreas de actuación de la enfermería, genera sobrecarga de trabajo, favorece el estrés, la aparición de errores y de eventos adversos, factores que comprometen la seguridad del paciente^(29)^.

La promoción de acciones educativas desde la perspectiva de la educación continua y permanente y la organización de entornos seguros entre el equipo, para la discusión de casos con fundamentos teóricos de la supervisión de enfermería, en consonancia con los conocimientos técnicos, así como reuniones periódicas entre los enfermeros, con el fin de mejorar la práctica profesional, intercambiar experiencias sobre cuestiones administrativas y organizativas, estableciendo entornos para la construcción colectiva, incentivando la fuerza interna del equipo, se consideraron factores potenciadores de la supervisión de enfermería en este contexto de trabajo. Un entorno de apoyo entre los profesionales para el intercambio de experiencias, la comunicación y la promoción de formaciones sobre temas pertinentes al contexto es una contribución positiva para el equipo en su conjunto y, en consecuencia, una mejora de la cualificación de la atención. Este apoyo incluye protocolos y técnicas de asistencia, limpieza, uso de equipos y tecnologías relacionadas con la atención directa, así como discusiones de casos después de los incidentes, ya presentes en el servicio, que tienen en cuenta las reflexiones sobre las situaciones vividas.

El apoyo a la inclusión de actividades educativas (cursos y formaciones), realizadas en horario laboral, sin perjuicio para el trabajador y con una dotación adecuada de profesionales para cubrir el turno de trabajo, podría ser una forma de afrontar los retos inherentes a la práctica de la supervisión de enfermería en el SAMU. A partir del análisis de las oportunidades de mejora, es posible impulsar el desarrollo de las competencias de los profesionales mediante la aplicación práctica de los conocimientos^(30)^.

La categoría «Regulación de plazas» concentró el mayor número de referencias negativas, lo que representa una limitación para la supervisión de enfermería. Los incidentes relacionados con la central de regulación de plazas pueden entenderse como complejos, ya que, aunque son una interfaz crucial entre el sistema y los servicios de salud con los usuarios y los profesionales, a menudo desencadenan ruidos de comunicación y conflictos que repercuten en la satisfacción y la adecuación de la atención al usuario, en las atribuciones y responsabilidades de diferentes puntos de la red de atención y en la actuación de los respectivos equipos de salud. En particular, en el caso de la enfermería, se puede inferir que representa la dimensión de la articulación política de la supervisión.

Los profesionales del SAMU dependen directamente de la orientación y el buen funcionamiento de la Central de Regulación de Urgencias y Emergencias (CRUE) para realizar su trabajo; por otra parte, no tienen visibilidad sobre la disponibilidad de plazas y todo el contexto sanitario de la región en el momento de la atención. Por lo tanto, es necesario establecer una relación de confianza con el fin de promover una atención ágil, segura y eficiente al paciente, minimizando los daños a la salud.

Algunos usuarios del SAMU desconocen o no han sido informados sobre el servicio de salud; estos deben buscar la razón por la que optan por la atención que consideran adecuada para el problema que enfrentan en ese momento. Es habitual el uso de este servicio como medio de transporte, para resolver demandas sociales o como puerta de entrada al SUS, fuera de situaciones de urgencia, ya que es un servicio en el que el usuario encuentra rápidamente un equipo completo de profesionales, tratamiento inmediato y servicios de mayor complejidad^(22)^.

Lo anterior pone de manifiesto aspectos macro organizativos que también repercuten en la supervisión de la enfermería; por ejemplo, ruidos en la interfaz con la CRUE, que pueden causar insatisfacción entre los profesionales, dar lugar a conflictos y retrasos en la atención, condiciones que no dependen de la actuación directa de los profesionales del SAMU. En este sentido, las estrategias de enfrentamiento no pueden ser simplistas; sin embargo, cabe destacar, de manera oportuna, la capacidad de utilizar estrategias educativas tanto con el equipo de enfermería para mitigar los problemas de comunicación y los conflictos, como la oportunidad de que el equipo informe a la población sobre el uso del SAMU y otros servicios de salud.

Como limitación del estudio, cabe destacar el análisis de un solo servicio, lo que restringe la posibilidad de ampliar el análisis del objeto de estudio; además, en ese momento, se estaba comenzando a flexibilizar las medidas de seguridad relacionadas con la pandemia de Covid-19 y se estaba llevando a cabo un proceso de reajuste de los servicios de salud. Sin embargo, a pesar de las limitaciones, el estudio contribuye significativamente al área de la enfermería, al destacar aspectos que limitan y potencian la supervisión de enfermería, ofreciendo *insights* valiosos para la mejora de la práctica profesional y la creación de un entorno de aprendizaje y diálogo. Por último, no se pretende proponer una guía o un modelo de trabajo, pero los resultados permiten sugerir aspectos que deben tenerse en cuenta en el enfoque de la supervisión de enfermería en el SAMU, como la posibilidad de explorar la práctica de la supervisión directa siempre que sea posible, con el enfermero como miembro del equipo de apoyo básico junto al técnico de enfermería.

A partir de los resultados y la discusión, se presenta una síntesis de los aspectos que potencian la realización de la supervisión de enfermería en el SAMU: el apoyo institucional en lo que se refiere a aspectos organizativos, como el vehículo de transporte, los materiales de limpieza y el mantenimiento, y en lo que se refiere a los instrumentos de ajuste para satisfacer las demandas de emergencia, como las horas extras; el apoyo a la inclusión de actividades educativas en el horario de trabajo, sin perjuicio para el trabajador, utilizando la educación como herramienta de supervisión de enfermería, a partir de un «*feedback»* oportuno; el fomento de un ambiente de apoyo entre los profesionales, con el fin de estimular el intercambio de experiencias; el fomento de la discusión de los casos después de los incidentes, con el fin de reflexionar sobre las situaciones vividas, analizadas y basadas en conocimientos técnico-científicos y éticos; reuniones periódicas con el equipo, como oportunidades para el intercambio de conocimientos, con una comunicación clara, objetiva y horizontal, capaz de reflejar la valoración de los profesionales y sus funciones; dimensionamiento adecuado de la plantilla de profesionales para cubrir el horario de trabajo junto con la organización del servicio para las actividades cotidianas, a partir de herramientas como horarios de trabajo, protocolos y procedimientos operativos de rutinas de limpieza, verificación y reposición de materiales; estímulo y realización de la supervisión directa del enfermero durante la atención del equipo de SBV, siempre que sea posible, con el objetivo de apoyar, evaluar la práctica profesional y realizar las intervenciones necesarias durante la práctica o mediante medidas educativas después de la atención.

Teniendo en cuenta los resultados y el debate, se presenta a continuación un resumen de los factores que limitan el desempeño de la supervisión de enfermería en el SAMU: El propio proceso de trabajo orienta al equipo en función del tipo de atención, lo que repercute en la fragmentación del equipo, ya que, en las intervenciones del equipo de soporte vital básico (SVB), el técnico de emergencias y el enfermero no ocupan el mismo espacio, lo que dificulta la supervisión de enfermería en este servicio, ya que no hay posibilidad de evaluar y apoyar al equipo de enfermería en el momento de la asistencia al paciente. En cuanto a los aspectos organizativos, la inadecuación e insuficiencia de materiales, el mantenimiento de los equipos y los vehículos de apoyo no son competencia del enfermero, pero repercuten en condiciones que pueden suponer riesgos asistenciales y profesionales; también en este tema, la falta de organización de las actividades inherentes a la rutina, como la limpieza, la organización y la reposición de materiales, hace que el equipo trabaje de forma descoordinada y desmotivada, lo que repercute en la atención adecuada y segura de las incidencias. A estos factores se suman las dificultades de comunicación y los conflictos presentes tanto en el equipo como entre los organismos de apoyo.

## CONCLUSIÓN

A partir de los resultados obtenidos en esta investigación, fue posible identificar la predominancia de aspectos que limitan el desempeño de la supervisión de enfermería en el contexto del SAMU. Esto destaca la urgencia de prestar más atención, particularmente en lo que respecta a la supervisión de enfermería, a los requisitos críticos necesarios para el desempeño de esta actividad y al enfoque educativo insuficiente dedicado a este componente gerencial, en el contexto estudiado.

Los resultados estimulan la reflexión sobre el desempeño del papel del enfermero en el ejercicio de la supervisión, pero también revelan aspectos relevantes relacionados con la gestión de la atención en el SAMU. Este escenario contribuye significativamente a identificar y mejorar los puntos críticos, proporcionando apoyo al enfermero en lo que se refiere a la práctica de la gestión del servicio, la gestión del equipo y la prestación de la asistencia. En este contexto, aunque los resultados no sean generalizables, es plausible que otras unidades en contextos similares puedan extraer beneficios de las investigaciones presentadas en este estudio.

Queda mucho por avanzar en los conocimientos teórico- prácticos sobre el ejercicio de la supervisión de enfermería en el SAMU, especialmente desde la perspectiva educativa, que, aunque se señala como un recurso utilizado, todavía presenta un potencial limitado.

## DISPONIBILIDAD DE DATOS

El conjunto de datos que respalda los resultados de este estudio no está disponible públicamente.
